# The route to transcription initiation determines the mode of transcriptional bursting in *E. coli*

**DOI:** 10.1038/s41467-020-16367-6

**Published:** 2020-05-15

**Authors:** Christoph Engl, Goran Jovanovic, Rowan D. Brackston, Ioly Kotta-Loizou, Martin Buck

**Affiliations:** 1 School of Biological & Chemical Sciences, Queen Mary University of London, London, E1 4NS UK; 2 Faculty of Medicine, Department of Medicine, Imperial College London, London, SW7 2AZ UK; 3 Faculty of Natural Sciences, Department of Life Sciences, Imperial College London, London, SW7 2AZ UK; 4 Present Address: Institute of Molecular Genetics and Genetic Engineering, University of Belgrade, Vojvode Stepe 444a, 11042 Belgrade, Serbia

**Keywords:** Bacterial genetics, Transcription

## Abstract

Transcription is fundamentally noisy, leading to significant heterogeneity across bacterial populations. Noise is often attributed to burstiness, but the underlying mechanisms and their dependence on the mode of promotor regulation remain unclear. Here, we measure *E. coli* single cell mRNA levels for two stress responses that depend on bacterial sigma factors with different mode of transcription initiation (σ^70^ and σ^54^). By fitting a stochastic model to the observed mRNA distributions, we show that the transition from low to high expression of the σ^70^-controlled stress response is regulated via the burst size, while that of the σ^54^-controlled stress response is regulated via the burst frequency. Therefore, transcription initiation involving σ^54^ differs from other bacterial systems, and yields bursting kinetics characteristic of eukaryotic systems.

## Abstract (web summary)

Transcription noise in bacteria is often attributed to burstiness, but the mechanisms are unclear. Here, the authors show that the transition from low to high expression can be regulated via burst size or burst frequency, depending on the mode of transcription initiation determined by different sigma factors.

## Introduction

Transcription is a series of discrete interactions of transcription factors and RNA polymerase with the promoter alongside any biochemical steps that these factors undertake^1,2^. As a consequence, transcription is stochastic^3,4^ and may follow either a Poisson distribution —mRNA is synthesized in random, uncorrelated events, with a uniform probability over time—or is described as bursty—mRNA is synthesised in episodes of high transcriptional activity^4–7^. Cell-to-cell variability (noise) within a population^3^ is increased through bursty transcription^4–7^. Noise underpins bacterial bet hedging whereby genetically identical cells display population-wide divergent phenotypes^8–11^. Bet hedging may offer a competitive advantage ensuring survival and is important in responses of bacteria to antibiotics, acquisition of drug-tolerant persistence and the use of cells harbouring non-native gene control circuits in synthetic biology^8–11^. Gene-specific and genome-wide sources of noise have been described^7^. However, the contributions of many molecular events involved in transcription—in particular those occuring during transcription initiation—are currently largely unknown^7,12^.

**Fig. 1:**
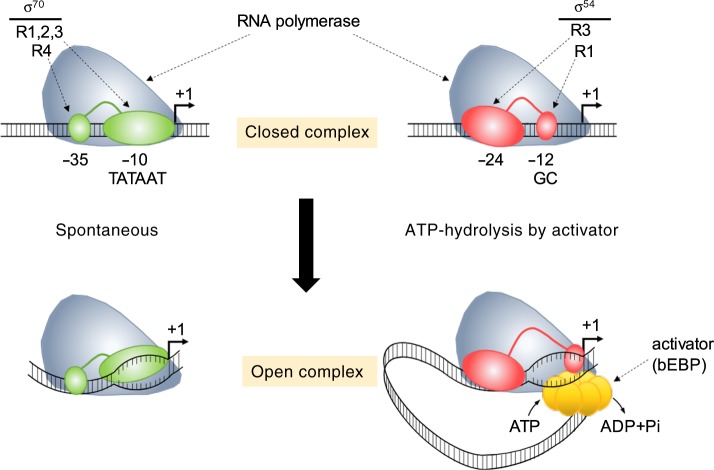
**Transcription initiation at σ**^**70**^**and σ**^**54**^**controlled promoters of bacteria**^1,2,13^. σ^70^ and σ^54^ direct RNA polymerase to bacterial promoters to form the closed complex. Regions (R) 1,2,3 and 4 of σ^70^ bind to DNA elements at position −10 (consensus sequence: TATAAT) and −35, while R1 and R3 of σ^54^ bind to sequences at position −12 (consensus sequence: GC) and −24 upstream of the transcriptional start site (+1). Open complex formation during transcription initiation occurs spontaneously at σ^70^ controlled promoters but requires the mechonchemical energy derived from ATP-hydrolysis by an activator, a bacterial enhancer binding protein (bEBP), at σ^54^ controlled promoters.

One major aspect of transcription initiation in bacteria is the need for a specificity factor termed sigma (σ) to direct RNA polymerase to the promoter^1,2,13^. Specificity factors comprise two distinct classes: the σ^70^ family combines all sigma factors that bind to −10/−35 promoter elements and in *E. coli* includes σ^70^, σ^19^, σ^24^, σ^28^, σ^32^ and σ^38^; in contrast, σ^54^ binds to −12/−24 promoter elements and forms a class of its own. Transcription initiation from σ^70^-dependent promoters involves the spontaneous isomerisation of the closed RNA polymerase-promoter complex to the open complex^1,2,14^ (Fig. 1). In marked contrast, open complex formation on σ^54^-dependent promoters strictly requires the action of cognate transcription factors, activators termed bacterial Enhancer Binding proteins (bEBP), for the promoter DNA opening event^13,15–19^ (Fig. 1). Significantly, to date all investigations regarding transcriptional noise and bursting within bacteria have centred solely on σ^70^-dependent promoters. It is however essential to establish a more global view given the similarity of σ^54^-dependent transcription to eukaryotic systems^20,21^ and coupled with the fact that σ^54^ is critical for many major bacterial adaptation strategies^22–27^. Understanding noise and bursting during σ^54^-dependent gene expression will enable determination of how heterogeneously these stress-related phenotypes are established across a cell population and where noise and bursting arises within the transcription time series. Since σ^54^ drives stress-induced gene expression, noisy and/or bursty behaviour may be advantageous, but to what extent (if any) and how it occurs is currently unknown.

In order to evaluate the impact of σ-factors in cell-to-cell variability, we analyse two paradigmatic stress responses, *Suf* and *Psp*, each dependent upon a contrasting σ-factor class. One response manages stress arising from exposure to oxidants^28^ and iron starvation^29^, the other manages membrane stress^23,27^.

## Results

### Determining transcriptional noise and burst kinetics

**Fig. 2:**
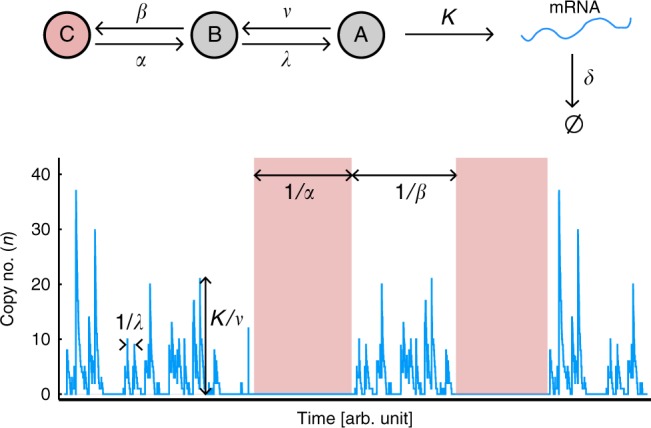
**Model of transcription used in this study**. We consider a multi-state stochastic model in which the promotor transitions between a deep inactive state (C), a primed inactive state (B) and an active state (A) in which transcription can occur. Slow switching between states C and B leads to an abundance of cells with no expression level. Rapid switches between states B and A lead to bursty transcription, in which bursts of average size *K*/*v* occur at an average frequency *λ*/*δ*.

We used RNA fluorescence in situ hybridization^30^ and the Spätzcells software^30^ (Supplementary Figs. 1–5) to determine the distribution of mRNA numbers per cell across bacterial populations(Supplementary Figs. 6, 7). We then calculated transcriptional noise (squared coefficient of variation; CV^2^)^31^ and burstiness (Fano factor; *F*)^32^. A Fano factor value of 1 corresponds to Poisson distribution (non-bursty), while Fano greater than 1 indicates bursty transcription. We also elaborated a model to provide a conceptual framework for interpreting our data. In the two-state Telegraph model of transcription^33–35^ the promoter transitions between an active and inactive state at rates *λ* and *ν* respectively. Transcription only occurs in the active state with a mean rate *Κ*, while mRNA decays at a rate *δ*. Transcription is bursty if *ν ≫ λ* and *Κ, ν ≫ δ* whereby bursts occur at an average frequency of *λ/δ* and an average size of *Κ/ν*. In line with previous propositions^36^ we considered that there may be multiple (nested) regulatory mechanisms at play, operating at different timescales to each other. This leads to a model in which there are multiple states of the transcription apparatus, only one of which is active in initiation (Fig. 2). Our model consists of having switching rates *α* and *β* between a “deep” inactive state (C) and a primed but inactive state (B). A single active state (A) then exists from which transcription can occur. If switching between states (C) and (B) is relatively slow, this leads to the behaviour displayed in Fig. 2, in which the gene experiences periods of complete inactivity, interspersed with periods of bursty transcription. In such a situation, the probability distribution for the copy number of mRNA at steady state can be shown to be well approximated by a zero-inflated negative binomial^30,37,38^ expressed mathematically as:

1 $$P\left( n \right) = \left\{ {\begin{array}{*{20}{l}} {\omega + \left( {1 - \omega } \right)\left( {1 - p} \right)^r,} \hfill & {{\mathrm{for}}\,n = 0} \hfill \\ {\left( {1 - \omega } \right)\left( {\begin{array}{*{20}{c}} {n + r - 1} \\ n \end{array}} \right)\left( {1 - p} \right)^rp^n,} \hfill & {{\mathrm{for}}\,n \, > \, 0} \hfill \end{array}} \right.$$

Here, *ω* refers to the fraction of time in which the gene is in the deep inactive state (C), and is related to the parameters by *ω* *=* *β/(α* *+* *β)*. This fraction is also the proportion of cells within a measured sample that are in this deeply inactive state. The parameters of the Negative binomial are related to the model parameters via *r* *=* *λ/δ*, and *p* *=* *ν*/(*ν* + *Κ*).

We then performed parameter estimation via model fitting. To infer parameters we used Bayesian inference via a custom Markov chain Monte Carlo (MCMC) algorithm, in order to obtain the probability distribution of these parameters, in light of the experimental data (Supplementary Figs. 8–10). This approach yielded not only the optimal parameters but also the degree of uncertainty regarding the inferred values. By performing this parameter inference separately for each set of data we obtained each of *r, p, ω* and consequently burst frequency *λ/δ* and burst size *Κ/ν*. Further details are given in the methods section.

### Noise and bursting in the σ^70^ controlled stress response

**Fig. 3:**
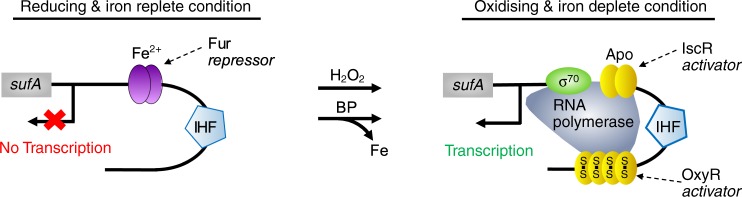
**Regulation of transcription of the σ**^**70**^**controlled*Suf*system**. The *Suf* system enables the assembly of Fe-S clusters of bacteria under oxidative stress and iron limitation^28,29,40–43^. Under reducing and iron replete conditions, transcription from the *Suf* promoter is repressed by Fur[Fe^2+^], preventing access of RNA polymerase/σ^70^ and the activator IscR to the promoter. The reduced form of the activator OxyR under these conditions has low affinity for the promoter. Under oxidising and iron deplete conditions (e.g. in presence of H_2_O_2_ and bipyridyl, BP), the oxidsed form of OxyR[S-S] and the Apo form of IscR have higher affinity for the promoter than the oxidised and/or Apo form of Fur. Integration Host Factor (IHF) bends the promoter DNA bringing OxyR[S-S] and RNA polymerase/σ^70^ into close contact. OxyR[S-S] and the Apo form of IscR stabilise the contact of RNA polymerase/σ^70^ with the promoter resulting in transcription.

The regulation of σ^70^ controlled promoters is often complex, responding to more than one signal through the action of multiple transcription factors with opposing effects on gene expression^1,2^. This characteristic distinguishes σ^70^ from σ^54^ controlled systems which only respond to one signal, typically through the action of a single transcriptional activator. Much of our knowledge on transcriptional noise and bursting of σ^70^ controlled systems stems from promoters that respond to signals that do not elicit a stress response and that are regulated via simple repression-activation (e.g. the *lac* promoter in *E. coli*)^4–7,39^. We investigated the expression profile of the σ^70^ controlled *Suf* system to establish whether the behaviour of stress responses under the control of a more complex regulation deviates from that of simpler adaptive systems such as lactose utilisation. S*uf* is encoded in a single operon (*sufABCDSE*) and enables the assembly of Fe-S clusters under oxidative stress and iron limitation^28,29^. Expression from the P_*sufA*_ promoter is induced in presence of hydrogen peroxide (H_2_O_2_) and/or the iron-chelator bipyridyl (BP)^28,29^. Regulation of P_*sufA*_ in response to these stressors involves the interplay between two activators (OxyR and IscR) and one repressor (Fur)^28,29,40–43^ (Fig. 3). Open complex formation during transcription initiation at σ^70^ controlled promoters such as P_*sufA*_ occurs spontaneously upon prolonged contact of RNA polymerase/σ^70^ with the promoter (Fig. 1). The role of the activators is to stablise the contact of RNA polymerase/σ^70^ with the promoter, while the repressor prevents access of RNA polymerase/σ^70^ (and in the case of P_*sufA*_ also of the IscR activator) to the promoter^28,29,40–43^ (Fig. 3).

We measured the level of gene expression, transcriptional noise and bursting of native P_*sufA*_ in wildtype and in cells lacking either *fur* (Δ*fur*) or *oxyR* (Δ*oxyR*), under unstressed basal conditions and in presence of H_2_O_2_ and BP applied singly and in combination. Cells lacking *iscR* consistently showed nearly no expression at any condition (data not shown), suggesting that IscR is the major regulator of P_*sufA*_, in line with previous findings^41,42^. The lack of expression meant that no further insight could be gained from this mutant in understanding the source of noise and burstiness of this promoter.

**Fig. 4:**
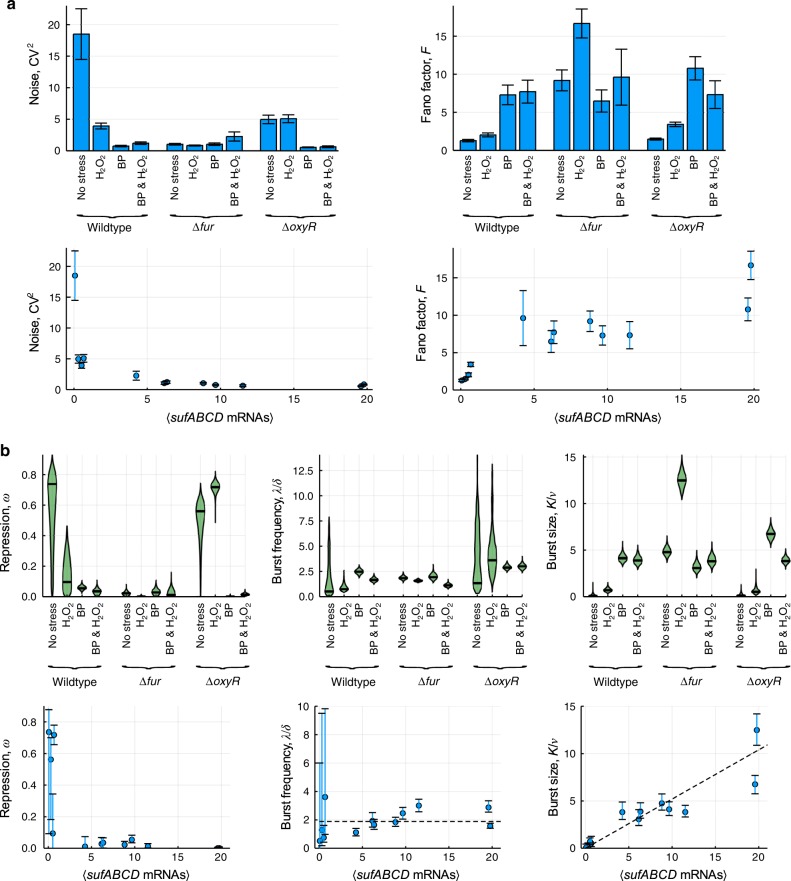
**Transcriptional noise and bursting of the σ**^**70**^**controlled*Suf*system**. Wildtype, Δ*fur* and Δ*oxyR E. coli* cells were grown in absence (no stress) and in presence of either H_2_O_2_, BP or BP+H_2_O_2_. The cells were then subjected to RNA fluorescence in situ hybridization with probes against *sufABCD* mRNA^30^. **a** Noise (CV^2^ = σ^2^/μ^2^) and burstiness (Fano factor *F* = σ^2^/μ) of *sufABCD* transcription under each condition (top) and as a function of the mean *sufABCD* mRNAs per cell (bottom). CV^2^ and *F* were calculated from the mean and standard deviations associated with the mRNA distributions in Supplementary Fig. 6. Data are presented as the statistic of the full set of data samples +/− the SEM obtained from *n* = 10,000 bootstrap resamples. **b** Burst kinetics of *sufABCD* transcription under each condition (top) and as a function of the mean *sufABCD* mRNAs per cell (bottom). Data are presented as the maximum a posteriori estimate (measure of centre of the error bar) and error bars are 95% credible intervals. Both are derived from the MCMC posterior distributions.

In wild-type cells under basal conditions, the mean *sufABCD* mRNA levels per cell are small (μ_Wildtype no stress_ = 0.07; σ_Wildtype no stress_ = 0.30) (Supplementary Fig. 6). Mathematically, the squared coefficient of variation is therefore large (CV^2^_Wildtype no stress_ = 18.50) (Fig. 4a) suggesting high expression noise. In light of the narrow mRNA distribution (Supplementary Fig. 6) we interpret this noise as occasional periods of low expression. The Fano factor and thus transcriptional bursting is low (*F*_Wildtype no stress_ = 1.29) (Fig. 4a) with a near-Poisson distribution of mRNAs across the population. This indicates a constant flux of (low level) mRNA production in absence of stress. Such a behaviour is typical for σ^70^ controlled promoters at low expression and for constitutive promoters^4–7,39^. Our data suggests that in P_*sufA*_ it is a consequence of repression and not activation, since lack of *fur* (*F*_Δ*fur*_ = 9.20) (Fig. 4a) but not of *oxyR* markedly increases the burstiness in absence of stress (*F*_Δ*oxyR*_ = 1.49) (Fig. 4a). In contrast, both Fur and OxyR contribute to the high levels of noise observed with wildtype in absence of stress since noise is reduced when either *fur* (CV^2^_Δ*fur*_ = 1.04) (Fig. 4a) or *oxyR* (CV^2^_Δ*oxyR*_ = 4.97) (Fig. 4a) is deleted.

As expected, in presence of stress and in absence of repression (Δ*fur*) the mean mRNA copy numbers per cell increase several fold (Supplementary Fig. 6). Our data is thereby in agreement with the reported competion for promoter binding at overlapping sites between the iron-bound and Apo forms of the repressor Fur and the activator IscR as the major regulatory mechanism of the P_*sufA*_ promoter, and an auxiliary role in regulation by H_2_O_2_ and OxyR in wildtype cells^28,29,40–43^. We observed stronger induction through iron chelation by BP and through deletion of Fur than through oxidative stress. H_2_O_2_ however became a stronger inducer than BP when it was applied singly in absence of repression by Fur (Supplementary Fig. 6). Interestingly, cells lacking *oxyR* showed stronger induction by BP and BP+H_2_O_2_ (Supplementary Fig. 6) than wildtype or cells lacking *fur*, suggesting that the presence of the second auxilliary activator has a somewhat dampening effect on transcription initiation by the major activator IscR. We further note that the mRNA levels produced per cell were consistently higher when the strong inducer BP was applied alone than in combination with the weaker inducer H_2_O_2_ (Supplementary Fig. 6).

Application of stress (singly or in combination) to cells that harboured the repressor (wildtype and Δ*oxyR*) reduced the noise (Fig. 4a) and increased the burstiness (Fig. 4a) of *Suf* transcription. The exception here was noise in absence of stress, which, as mentioned above, was already reduced in cells lacking OxyR and did not further decrease upon addition of H_2_O_2_. Thus, OxyR clearly plays a role in noise generation in absence of stress. However, OxyR appears to have no major effect on noise in presence of single stress (H_2_O_2_ as well as BP), since noise in wildtype and Δ*oxyR* under these conditions was largely the same. Generally, the levels of noise and burstiness correlate with the strength of the inducing signal. Application of the stronger inducer BP (singly and in combination with H_2_O_2_) resulted in stronger noise reduction and a stronger increase in burstiness than application of the weaker inducer H_2_O_2_. Interestingly, noise was slightly lower when BP was applied alone than in combination with H_2_O_2_. This was slightly more pronounced in Δ*fur* (CV^2^_Δ*fur*+BP_ = 1.05; CV^2^_Δ*fur*+BP+H2O2_ = 2.26) (Fig. 4a) than in wildtype (CV^2^_Wildtype+BP_ = 0.76; CV^2^_Wildtype+BP+H2O2_ = 1.22) (Fig. 4a), while in Δ*oxyR* we observed no difference in noise between BP and BP + H_2_O_2_ (CV^2^_Δ*oxyR*+BP_ = 0.55; CV^2^_Δ*oxyR*+BP+H2O2_ = 0.64) (Fig. 4a). Our data therefore implies that the combined action of two activators in presence of two inducing signals can increase the transcriptional noise of a σ^70^ promoter while the repressor can, to some degree, counteract this effect. When we compared the levels of noise and bursting with the mean number of *sufABCD* mRNAs per cell, it became clear that noise and burstiness are strongly correlated with the level of expression (Fig. 4a). Overall, the key determinant of noise and burstiness of P_*sufA*_ is the ability to repress transcription, given that noise is low and burstiness high under any condition when *fur* is absent. This is consistent with recent findings that cell-to-cell variability can be attributed to the action of a transcriptional repressor^44^.

Fitting our extended Telegraph model to the experimental data of mRNA distributions (red solid line, Supplementary Fig. 6) further enabled us to extract the parameters *ω*, *λ* and *K*/*ν* underpinning bursty transcription (Fig. 4b). The fraction of time and the proportion of cells (*ω*) in which the σ^70^ controlled promoter is in the deep inactive state (C) is linked to the repression by Fur, since the value of *ω* is high when Fur is operating (i.e. in wildtype cells in absence of stress) yet *ω* is always low when cells are lacking Fur (independent of whether or not the cells are also exposed to stress). Furthermore, the value of *ω* in presence of H_2_O_2_ showed a strong dependence on OxyR. Indeed, this was one of the strongest effects of OxyR in our entire data set. We thus conclude that a major role for OxyR in the regulation of P_*sufA*_ transcription is to reduce the time and proportion of cells in which P_*sufA*_ is in the deep inactive state and thus to prime P_*sufA*_ for expression under oxidative stress.

The burst frequency (*λ/δ*) of P_*sufA*_ was largely similar and did not scale with mean mRNA numbers per cell at higher levels of expression, i.e., when repression was removed e.g. through *fur* deletion or addition of BP (singly or with H_2_O_2_) in wildtype and Δ*oxyR* cells. We note however some modest burst frequency modulation at low expression in unstressed wildtype and Δ*oxyR* cells and also in Δ*oxyR* in presence of H_2_O_2_ (Fig. 4b), as was observed with the σ^70^-controlled P_*lac/ara*_ promoter at low inducer concentrations^4^.

In contrast, the size of transcriptional bursts (*K*/*ν*) from P_*sufA*_ was small at low expression (e.g. in unstressed wildtype cells). Burst size scaled however with signal strength and mean mRNA numbers per cell to yield large bursts at high expression (Fig. 4b). Notably, removal of *oxyR* had no major effect on burst size under any condition compared with wildtype (Fig. 4b).

Taken together, we conclude that the level of bursting during transcription from P_*sufA*_ is related to the level of expression and controlled via the burst size. Hence, despite being a more complex promoter and driving the expression of a stress response, P_*sufA*_ largely recapitulates the behaviour of simpler σ^70^ controlled promoters of stress unrelated adaptive responses^4–7,39^.

### Noise and bursting in the σ^54^ controlled stress response

After validating our technical approach and computational modelling via P_*sufA*_, we next established the sources of noise and bursting of the previously unexplored enhancer dependent σ^54^ dependent transcription.

**Fig. 5:**
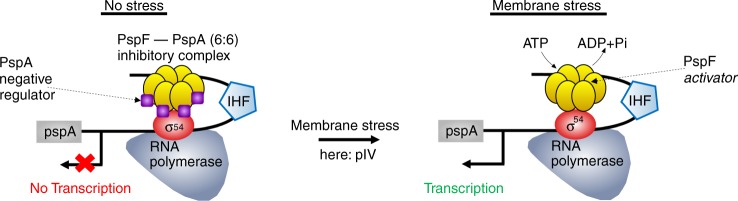
**Regulation of Transcription of the σ**^**54**^**controlled*Psp*system**. The *Psp* system stabilises damaged inner membranes of bacteria^23,27^. Transcription of *Psp* is controlled by RNA polymerase/σ^54^ and requires ATP hydrolysis by the bacterial enhancer binding and activator protein PspF. In absence of membrane stress, the negative regulator PspA (as low-order oligomer) forms an inhibitory complex with PspF in a 6:6 ratio. PspA prevents ATP hydrolysis by PspF and thus *Psp* transcription. IHF bends the promoter DNA bringing the enhancer-bound PspF in contact with the RNA polymerase/σ^54^. Membrane stress, e.g., through mislocalisation of proteins such as secretin pIV to the inner membrane enables the release of PspA from PspF. Subsequent ATP hydrolysis by PspF yields open complex formation and initiates *Psp* transcription. Additional information on the regulation of transcription of the σ^54^ controlled *Psp* system can be found in Supplementary Figs. 11, 12.

We chose one of the best studied σ^54^ controlled systems, the model stress response *Psp* which maintains proton motive force under membrane stress^23,27^ (Fig. 5). *Psp* is implicated in biofilm formation, virulence, and antibiotic persistence. A comprehensive overview of *Psp* regulation and function is reviewed in refs. ^23,27^.

**Fig. 6:**
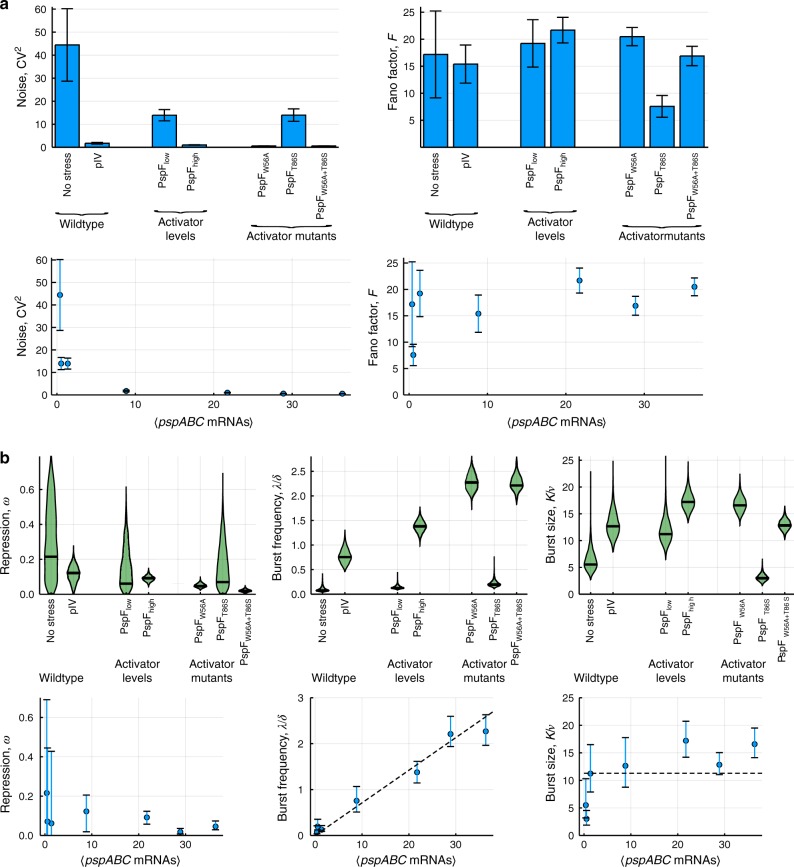
**Transcriptional noise and bursting of the σ**^**54**^**controlled*Psp*system**. Wildtype *E. coli* cells grown in absence (no stress) and presence of membrane stress by pIV; and *E. coli* cells expressing either low (PspF_low_) or high (PspF_high_) levels of native activator or low levels of activator variants with native activity subject to negative regulation (PspF_WT_), constitutive activity (PspF_W56A_), negatively regulated activity and reduced affinity for the closed complex of RNA polymerase/σ^54^ (PspF_T86S_), constitutive activity and reduced affinity for the closed complex of RNA polymerase/σ^54^ (PspF_W56A+T86S_). (**a**) Noise (CV^2^ = σ^2^/μ^2^) and burstiness (Fano factor *F* = σ^2^/μ) of *pspABC* transcription under each condition (top) and as a function of the mean *pspABC* mRNAs per cell (bottom). CV^2^ and *F* were calculated from the mean and standard deviatons estimated from the mRNA distributions in Supplementary Fig. 7. Data are presented as the statistic of the full set of data samples +/− the SEM obtained from *n* = 10,000 bootstrap resamples. **b** Burst kinetics of *pspABC* transcription under each condition (top) and as a function of the mean *pspABC* mRNAs per cell (bottom). Data are presented as the maximum a posteriori estimate (measure of centre) and error bars are 95% credible intervals. Both are derived from the MCMC posterior distributions.

Initially we measured mRNA copy numbers arising from the native P_*pspA*_ promoter in wildtype with and without stress, here pIV secretin^23^. Under basal conditions (no stress), the mean mRNA levels per cell produced from P_*pspA*_ are low (μ_Wildtype no stress_ = 0.39; σ_Wildtype no stress_ = 2.58) (Supplementary Fig. 7). Gene expression is noisy (CV^2^_Wildtype no stress_ = 44.44) (Fig. 6a) and bursty (*F*_Wildtype no stress_ = 17.19) (Fig. 6a). This basal level of noise and burstiness is significantly higher in the σ^54^ than in the σ^70^ controlled system, presumably reflecting their differing dependencies for open complex formation. Under stress, mean mRNA copy numbers per cell increase several fold, (μ_Wildtype+pIV_ = 8.84; σ_Wildtype+pIV_ = 11.67) (Supplementary Fig. 7) whilst noise is markedly reduced (CV^2^_Wildtype+pIV_ = 1.74) (Fig. 6a); strikingly however, and in marked contrast to σ^70^, burstiness remains largely unchanged (*F*_Wildtype+pIV_ = 15.40) (Fig. 6a) compared with unstressed conditions.

Recall that transcription initiation from σ^54^-dependent promoters strictly requires the mechanochemical action of an enhancer binding transcriptional activator (here PspF)^15,16^ (Fig. 1, Supplementary Fig. 11). Consequently, we failed to detect any measurable activity from the P_*pspA*_ promoter in cells lacking *pspF* both in the absence and presence of stress (data not shown).

To unravel the precise contribution of the activator, we examined noise and burstiness of the promoter by altering key parameters that regulate activator function. Previous studies have shown that the cellular levels of PspF are low and remain constant before and after stress^45^. This is due to autoregulation via a negative feedback exerted by PspF on its own expression^45^ (Supplementary Fig. 12). Under basal unstressed conditions, the activator is inhibited through negative regulation^23,46,47^. In the *Psp* system, it is achieved through direct protein–protein interaction between PspF and its negative regulator PspA^23,46,47^ (Fig. 5). Under stress, PspA associates with the innner membrane and binding to PspF is diminished enabling PspF to activate transcription. Note that PspF, unless bound by PspA, is constitutively active for driving open complex formation^47^.

We first explored how varying the activator levels and removing autoregulation affects the noise and burstiness of σ^54^-dependent transcription. We achieved this through inducible heterologous control of PspF expression. At high PspF levels there is not enough PspA present to bind to and therefore inhibit all of the PspF. The outcomes of high level expression of PspF are similar to those between unstressed and stressed wildtype conditions. Between low and high levels of PspF, we observed a several fold increase in mRNA copy numbers (Supplementary Fig. 7) and a marked reduction in noise (Fig. 6a), while levels of burstiness were unchanged (Fig. 6a). We note however that expression of P_*pspA*_ in unstressed wildtype cells was markedly noisier (CV^2^_Wildtype no stress_ = 44.44) (Fig. 6a) than at low level PspF overexpression (CV^2^_PspF_low_ = 13.92) (Fig. 6a). We propose that this is a consequence of the autoregulation that greatly limits the number of PspF molecules in the wildtype system. This limitation is absent due to heterologous control of PspF levels in the activator overexpression experiment.

Next we altered the efficiency of the activation step. In σ^54^-dependent promoters, activation (and hence open complex formation) is a multi-step process that involves (i) ATP hydrolysis (inhibited by negative regulation) and (ii) sustained contact of the activator with the closed complex (RNA polymerase/σ^54^/promoter DNA)^13,15–19^ (Figs. 1, 5, Supplementary Fig. 11).

We utilised a variant form of the activator PspF (PspF_W56A_) expressed at low levels which is unable to interact with the negative regulator PspA and thus escapes its inhibitory function^46–49^ (Supplementary Figs. 5, 11). Under this condition, all PspF present within the cell albeit at low levels will be constitutively active. In this way, this condition should resemble the wildtype scenario in presence of stress. Similar to the effect of activator overexpression, with this variant we observed substantially higher mRNA copy numbers (μ_PspF_W56A_ = 36.40; σ_PspF_W56A_ = 27.31) (Supplementary Fig. 7) and lower noise (CV^2^_PspF_W56A_ = 0.56) (Fig. 6a) than in the native system under stress (μ_Wildtype+pIV_ = 8.84; σ_Wildtype+pIV_ = 11.67; CV^2^_Wildtype+pIV_ = 1.74). We presume that these differences arise from some residual negative regulation that occurs in the native system under stress. Strikingly however, removal of negative regulation of the activator has no marked effect on the burstiness of the σ^54^ controlled promoter (Fig. 6a). Following on from this, we examined how weakening the contact between the activator and the closed complex affects transcriptional noise and bursting of σ^54^ controlled promoters. To do this, we utilised a variant form of PspF (PspF_T86S_) that has reduced affinity for the closed complex formed by RNA polymerase and σ^54^ at the promoter^50,51^(Supplementary Figs. 5, 11). It is important to note that this form of PspF retains a native ATP hydrolysis activity and is still subject to negative regulation by PspA^50,51^. With this mutant, mRNA copy numbers are slightly reduced (μ_PspF_T86S_ = 0.54; σ_PspF_T86S_ = 2.03) (Supplementary Fig. 7) while noise (CV^2^_PspF_T86S_ = 13.96) (Fig. 6a) is similar to that observed with the wildtype activator control PspF_low_ (CV^2^_PspF_low_ = 13.92). Notably however, the promoter is markedly less bursty in presence of PspF_T86S_ (F_PspF_T86S_ = 7.57) (Fig. 6a) than under any other condition tested. When we increased the availability of this weakend activator variant by removing the negative regulation (PspF_W56A+T86S_), noise (CV^2^_PspF_W56A+T86S_ = 0.58) (Fig. 6a) returns to PspF_W56A_ levels (CV^2^_PspF_W56A_ = 0.56) (Fig. 6a). The mRNA copy numbers per cell are slightly reduced (μ_PspF_W56A+T86S_ = 28.87; σ_PspF_W56A+T86S_ = 22.08) (Supplementary Fig. 7) while burstiness (*F*_PspF_W56A+T86S_ = 16.89) (Fig. 6a) is largely similar compared with PspF_W56A_. We conclude that the reduction in burstiness seen with the T86S mutant under negative regulation (PspF_T86S_) is due to the inefficient remodelling of the closed to the transcriptionally active open complex. Taken together, the quality of the contact between the activator and the closed complex is a determinant of the burstiness but not the level of noise during transcription from σ^54^ controlled promoters.

We next explored whether transcriptional noise and bursting of the σ^54^ controlled promoter correlate with the level of expression as is seen with σ^70^ (Fig. 4a, b)^4–7,39,52^. Indeed, similar to σ^70^, transcriptional noise of σ^54^ controlled promoters decreases exponentially with increasing expression (Fig. 6a). Yet, the two sigma factors differ in their correlation between transcriptional bursting and the level of expression. Recall that burstiness of the σ^70^ controlled promoter is low, with near-Poisson mRNA production, at low expression (in absence of stress) and increases exponentially with increasing expression level (either induced by stress or by removal of the repressor) (Fig. 4a)^4–7,39^. In contrast, burstiness of the σ^54^ controlled promoter is high and largely unchanged (except for PspF_T86S_), indicating non-Poisson mRNA production at all levels of expression (Fig. 6a).

Model fitting to the experimental data (red solid line, Supplementary Fig. 7) further enabled us to extract the parameters underpinning transcriptional bursting (Fig. 6b). The fraction of time and the proportion of cells (ω) in which the σ^54^ controlled promoter is in the deep inactive state (C) correlates with the level of negative regulation of the activator. It is lowest in the W56A mutant where negative regulation is absent, ATP hydrolysis constitutively active and remodelling of the closed to the transcriptionally active open complex efficient due to the proficient contact of this constitutive activator with the closed complex.

**Fig. 7:**
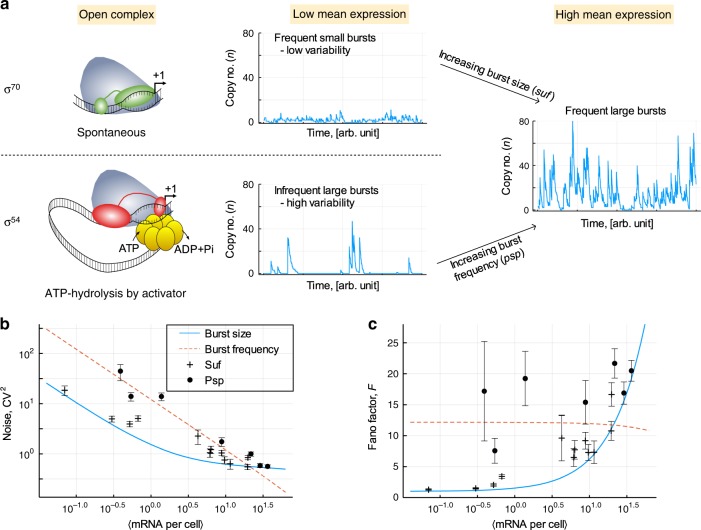
**Comparison of noise scaling and bursting mechanisms of σ**^**70**^**and σ**^**54**^**controlled transcription**. In bacteria, the form of transcriptional bursting is determined by the route to transcription initiation. We observe that for σ^70^ controlled promotors in which open complex formation occurs spontaneously, regulation occurs by relieving repression, corresponding to a reduction of the parameter *ν*. Low expression levels therefore consist of relatively frequent but small bursts that lead to low variability (**a**). For σ^54^ controlled promotors in which open complex formation requires ATP-hydrolysis dependent activation, regulation occurs by increasing the rate at which this activation occurs. Low expression levels in this case correspond to large but infrequent bursts, leading to large variability (**a**). At high expression levels, both types of promotor may converge towards similar behaviour. The relationships between mean expression level and the Noise (**b**) and Fano factor (**c**) can be expressed mathematically in each of these cases, provided that regulation is via only one of burst size or burst frequency. These expressions are provided in materials and methods and plotted in (**b**) and (**c**) along with the measured cell behaviour. Data are presented as the statistic of the full set of data samples +/− the SEM obtained from *n* = 10,000 bootstrap resamples.

Strikingly, burst size and frequency were in stark contrast to the expectations arising from previous studies of σ^70^ controlled promoters. The size of transcriptional bursts from P_*pspA*_ was large under any condition, including at low expression levels; although we note a modest increase in burst size upon release of negative regulation. Moreover, burst size was reduced when the contact of the activator to the closed complex is inefficient. This effect was particularly strong at low level of expression when the activator is negatively regulated (compare PspF_T86S_ with PspF_low_), but only modest at high level of expression in absence of negative regulation (compare PspF_W56A+T86S_ with PspF_W56A_) (Fig. 6b, Supplementary Figs. 7, 11). In contrast, the frequency by which P_*pspA*_ generates transcriptional bursts scaled with the level of negative regulation of activator ATP hydrolysis and thus with gene expression (Fig. 6b). Burst frequency was low at low expression in unstressed wildtype cells and at low activator levels (PspF_low_). It increased in wildtype in presence of stress as well as at high activator levels (PspF_high_), in both cases some low level negative regulation still occurs. Yet, burst frequency was highest when negative regulation was completely abolished in the W56A and W56A + T86S mutant. The efficiency of the contact between activator and the closed complex however appears to have no major effect on the burst frequency. We discounted that our observations were simply due to differences in (i) gene dosage (Supplementary Fig. 13, Supplementary Note 1) or (ii) RNA lifetime (Supplementary Note 1). Taken together, σ^54^-dependent transcription is always bursty and the transition from low to high expression is controlled via the burst frequency and not via the burst size (Fig. 7). To our knowledge, this is the first time such a behaviour has been observed with a bacterial promoter^4–7,39^; indeed it rather resembles the behaviour of promoters from yeast and higher organisms^4,53–56^. Our study therefore fundamentally challenges and extends the current view and understanding of transcriptional noise in bacterial gene expression.

## Discussion

Although it is well established that noise and bursting is a fundamental property of inducible gene transcription, a precise picture of the underlying molecular events has not yet emerged. It is implied that domain specific differences in burst kinetics exist^4^ given that in contrast to other domains, in bacteria burstiness as well as burst size increased with higher expression in all promoters studied to date. This was further interpreted as being indicative of a global control of transcriptional bursting. Contradictory to this idea of gene-independent determinants of burst kinetics, it was shown that burstiness and burst size can be modulated by changing the efficiency of transcription regulation of an individual bacterial gene^39^.

A fuller understanding of the sources of transcriptional noise and bursting in bacteria requires an appreciation of the biochemical complexity of transcription regulation to avoid oversimplified views of the events that lead to open complex formation during transcription initiation. The impact of transcription initiation on noise and bursting has been acknowledged^57,58^. Yet, the contribution of sigma factors—whose role is to facilitate transcription initiation—has so far been overlooked. All bacterial promoters studied to date for noise and bursting are controlled by σ^70^. However, the evolutionarily distinct σ^54^ drives a fundamentally different mode of open complex formation. Whether or not the two sigma factor classes differ in the generation of noise and bursting is currently unknown. Yet, such knowledge is critical in order to gain a better appreciation of the scope of noisy and bursty transcription displayed by bacteria. It also enables a better understanding of the mechanisms underpinning cell-to-cell variability and thus phenotypic variation of σ^54^ controlled adaptive responses, e.g., biofilm formation, nitrogen fixation, virulence or antimicrobial persistence; such an understanding is essential when aiming to manipulate beneficial and pathogenic interactions of bacteria with their human, animal and plant hosts.

The present study demonstrates that in bacteria the class of sigma factors, and as such the route to open complex formation during transcription initiation, determines the level and kinetics of transcriptional bursting. Low mean expression results from frequent small transcription bursts with low variability at σ^70^-dependent promoters or from infrequent large bursts with high variability at σ^54^-dependent promoters (Fig. 7). High mean expression resulting from frequent large transcription bursts is achieved by decreasing ν and thus increasing the burst size (*Κ*/*ν*) at σ^70^-dependent promoters or by increasing the burst frequency *λ/δ* at σ^54^-dependent promoters (Fig. 7). Hence, transcription from σ^70^-dependent promoters is altered via the number of mRNAs produced in the active state, and not via the rate of transition between the primed inactive and the active state. This is consistent with spontaneous unregulated promoter opening and regulated promoter access of RNA polymerase, as observed for σ^70^ factors. Transcription from σ^54^-dependent promoters, however, is altered via the rate of transition between the primed inactive and the active state, while the number of mRNAs produced in the active state remains unchanged. This is consistent with regulated promoter opening (requiring ATP-hydrolysis by an enhancer binding activator) and unregulated access of RNA polymerase to the promoter, both hallmarks of transcription initiation involving σ^54^. Strikingly, a similar behaviour is also observed in enhancer-dependent transcription in mamalian cells^**56**^. This implies that the type of regulation and not domain-specific constraints determine the transcriptional burst kinetics of a gene.

While the two sigma factor classes yield opposing burst kinetics, noise (cell-to-cell variability of transcription within a population) simply correlates with the level of gene expression irrespective of the sigma factor. Our results imply that under basal and stress conditions bacteria utilise a universal adaptive behaviour. Prior to stress, transcription is noisier and hence cells are more heterogeneous; presumably reflecting opportunity to bet hedge. Upon stress perception, transcriptional noise is reduced through elevated gene expression yielding a more homogeneous cell population. Our observations therefore suggest a mixed strategy to cope with stress, using both environmental sensing and bet hedging. Environmental sensing is arguably more cost effective in that gene expression only occurs in presence of stress. Here we assume the low level expression of the sensory apparatus before stress is less costly than the full stress response. Examples where this may not be so can be envisaged, but is not the case for those studied here. The applicability of environmental sensing however is limited to stress conditions that are neither too sudden nor too severe given the phenotypic lag between sensing and responding. Bet hedging (gene expression in absence of stress) may be more costly but eliminates the phenotypic lag and thus provides an escape route for sudden and severe stress by priming the cells for less favourable conditions. This strategy may represent an adaptation to more stringent environmental pressure in line with the idea that gene expression noise may be subject to evolutionary selection^59^.

## Methods

### Bacterial strains and growth conditions

All experiments were performed with *Escherichia coli* K12 MG1655 strains. Genes were deleted by P1 transduction using strains from the KEIO collection of *Escherichia coli* mutants^60^ as donors or by lambda red recombineering^61^. Strains were typically grown in Luria-Bertani (LB) broth or on agar at 37 °C. For RNA fluorescence in situ hybridization, strains were grown in M9 minimal medium (Teknova) at 37 °C, from an inoculum of 150 μl from an LB overnight culture to a final OD_600_ of 0.4 in a culture volume of 30 ml in a 250 ml flask. The bacterial cultures were supplemented as required with antibiotics at the following concentrations: chloramphenicol 30 μg/ml; kanamycin 25 μg/ml. Exposure to isopropyl β-D-1-thiogalactopyranoside (IPTG) at 1 mM final concentration for 1 hour was used to express pIV from plasmid pGZ119EH. Arabinose 0.02–0.2% (^w^/_v_) and glucose 0.4–1% (^w^/_v_) were used to express PspF (PspF_low_, PspF_high_, PspF_WT_, PspF_W56A_, PspF_T86S_ and PspF_W56A+T86S_) from pBAD18-cm in cells lacking chromosomally encoded *pspF*.

### Fluorescent probes

Fluorescent DNA probes (purchased from LGC Biosearch Technology) to detect the mRNA of *sufABCD* and *pspABC* structural genes were designed using the Stellaris® Probe Designer version 4.2; the oligo length was set at 20 nt, the minimal spacing length at 2 nt and the masking level at 1–2. The probes were labelled by 6-carboxytetramethylrhodamine, succinimidyl ester (6-TAMRA).

### RNA fluorescence in situ hybridization

Bacterial cells were grown in M9 medium to an OD_600_ of 0.4 and collected by centrifugation. Cells were fixed in 1 ml of ice-cold 1× PBS in DEPC-treated water with 3.7% (^v^/_v_) formaldehyde (30 min incubation at room temperature), washed twice in 1 ml 1× PBS in DEPC-treated water, permeabilised in 1 ml 70% (^v^/_v_) ethanol in DEPC-treated water (1 h gently mixing at room temperature) and washed again in 1 ml 2× SSC in DEPC-treated water with 40% (^w^/_v_) formamide. Hybridisation was performed through incubation of the cells overnight at 30 °C in hybridisation buffer (2x SSC in DEPC-treated water, 40% (^w^/_v_) formamide, 10% (^w^/_v_) dextran sulfate, 2 mM ribonucleoside-vanadyl complex, 0.2 mg/ml BSA and 1 mg/ml carrier *E. coli* tRNA) with 1 μM of the appropriate fluorescent probe(s). Subsequently, 10 μl of cells in hybridisation buffer were washed twice in 200 μl 2× SSC in DEPC-treated water with 40% (^w^/_v_) formamide and incubated for 30 min at 30 °C. Chromosomes were stained by incubating the cells for 30 min at 30 °C with 10 μg/ml DAPI (4′,6-diamidino-2-phenylindole) in 200 μl 2× SSC in DEPC-treated water with 40% (^w^/_v_) formamide. The cells were washed again in 200 μl 2× SSC in DEPC-treated water with 40% (^w^/_v_) formamide and resuspended in 10 μl 2× SCC in DEPC-treated water. For imaging, 2 μl of the cell suspension were immobilized using 1% (^w^/_v_) agarose pads on 35 mm, high μ-Dishes (ibidi).

### Microscopy to determine mRNA copy number per cell

A Zeiss Axio Observer widefield microscope with LED illumination was used to acquire images from multiple fields of view for all required channels (brightfield, DAPI, Cy3 for 6-TAMRA) (Supplementary Figs. 1–5). Image stacks with 200 nm intervals between successive z-slices were captured and converted to TIFF format using ImageJ^62^. Cell segmentation masks from brightfield or DAPI images were generated via Schnitzcells^63^ in MATLAB (MathWorks). The mRNA copy number per cell was determined via Spätzcells^30^ in MATLAB (MathWorks) using images acquired through the Cy3 (6-TAMRA) channel in combination with the cell segmentation masks. Fluorescent spots within selected cells were detected automatically and differentiation of specific from nonspecific probe binding was achieved by selecting a false-positive threshold using non-expressing cells (Δ*sufABCD*, Δ*pspABC*, Δ*pspF*). The probability distribution of peak height and intensity of fluorescent spots was extracted (Supplementary Figs. 1–5). The threshold to discard false positive spots was determined via the 99.9 percentile of spots in non-expressing cells. The spot intensity distribution from a low expressing strain was fitted to a multiple Gaussian function (Supplementary Figs. 1, 4, 5). The spot intensity of a single mRNA was determined through the mean of the first Gaussian. The number of mRNA molecules in each cell was extracted via the spot intensity of a single mRNA. The data was used to calculate the probability distribution, mean and standard deviation of the mRNA copy number per cell at the population level (Supplementary Figs. 6, 7).

### Mathematical modelling and computational analysis

We calculated noise, burstiness and burst kinetics from a population of cells expressing up to 150 mRNAs of the analysed genes. This is based on the available in vitro data on the activity of σ^54^ promoters showing that each open promoter complex takes 1–2 min to form^17^. During one cell division (~30 min) we assume therefore that 50 mRNAs per cell would be towards the top end of what might accumulate if the usual parameters of promoter activity and mRNA stability were being met. We note that some cells however appeared to accumulate several hundred target mRNAs. We propose that these high content mRNA cells are imaging or hybridisation artefacts e.g. through probe aggregation or non-specific genome-wide hybridisation. These cells were therefore omitted from the analyses presented in the main text. For completenesss however, we have included the data derived from the analyses including these cells (Supplementary Fig. 14). The overall trends of noise, burstiness and burst kinetics were similar to the range of 0–150 mRNAs per cell.

Model fitting was performed via a Bayesian inference approach using MCMC sampling. Given the equation for the probability distribution provided by the model, we evaluated the likelihood of obtaining a given set of data (D). If the copy number of mRNA in the *i*’th cell is denoted n_*i*_ with *M* cells in total, the likelihood of obtaining this data, given a particular set of parameters, is:

2 $$L\left( {D{\mathrm{|}}\theta } \right) = \mathop {\prod }\limits_{i = 1}^M P(n_i|\theta ).$$

Here *θ* = [*ω*; *r*; *p*] is the vector of parameters that define the distribution. In a Bayesian framework we then evaluated the posterior distribution over these parameters according to:

3 $$P(\theta |D) \propto L(D|\theta )\pi (\theta );$$

where *π*(*θ*) are our prior distributions. Priors for *ω* and *p* were uniform (0,1) and therefore flat across the range of permissable values. The prior for *r*(=*λ*) was set as the half-Normal(*μ* = 0,*σ* = 20), truncated to positive values. This prior has minimal effect around the region of the inferred distribution, but ensures good convergence properties for the MCMC. A custom Metropolis-Hastings MCMC scheme was implemented to sample from the posterior distribution, the code for which is freely available. A multivariate Gaussian proposal distribution was used, in which the standard deviation was set at 5% of the current parameter values to ensure a reasonable acceptance ratio and good convergence. Chains were generally run to collect 500,000 samples with 100,000 dicarded as burn-in and thinning applied at a factor of 100. Each chain was examined for good convergence and restarted from the old chain when necessary. Outputs from the MCMC are displayed in Supplementary Figs. 9, 10.

### Prediction of noise and Fano factor

In the case that the mean expression level is determined by regulation of only one kinetic parameter, it is possible to determine the variance of the resulting copy number distribution as a function of the mean. This enables one to form expressions for the squared noise (CV^2^) and Fano factor (*F*) as a function of the mean expression level and the other (non-varying) kinetic parameters. Expressions for the Fano factor are provided through Eqs. (3) and (4) in ref. ^6^ and repeated here for completeness and consistency with our terminology. In each of the expressions below the degradation rate δ has been set equal to 1 and therefore omitted, since all parameters considered here are normalised with respect to δ.

If regulation is performed only via *λ*, and therefore the burst frequency, the following expression hold for each of the squared noise and Fano factor,

4 $${\mathrm{CV}}^2\left( \langle n \rangle \right) = \frac{1}{\langle n \rangle} + \frac{{\left( {K - \langle n \rangle} \right)^2}}{{\langle n \rangle\left( {\nu K + K - \langle n \rangle} \right)}},$$

5 $$F\left( \langle n \rangle \right) = 1 + \frac{{\left( {K - \langle n \rangle} \right)^2}}{{\left( {\nu K + K - \langle n \rangle} \right)}}.$$

Conversely, if regulation is only via ν, and therefore the burst size, the following expressions hold,

6 $${\mathrm{CV}}^2\left( \langle n \rangle \right) = \frac{1}{\langle n \rangle} + \frac{{K - \langle n \rangle}}{{\lambda K + \langle n \rangle}},$$

7 $$F\left( \langle n \rangle \right) = 1 + \frac{{\langle n \rangle\left( {K - \langle n \rangle} \right)}}{{\lambda K + \langle n \rangle}}.$$

In plotting the predictions of these equations for Fig. 7, one has to choose fixed values of the other parameters. We do this here by taking a relevant average of the inferred values. For the *Suf* data in which we infer variation in only the burst size, an average value of λ = 1.89 is obtained. For the *Psp* data, we take an average value of the ratio *K*/ν as 11.3. One must then still choose a value for *K*/*δ*, which here we set as 1000, although the results are not very sensitive to this choice.

### Reporting summary

Further information on research design is available in the Nature Research Reporting Summary linked to this article.

## Supplementary information

Supplementary Information

Reporting Summary

## Data Availability

The authors declare that the data supporting the findings of this study are available within the paper and its Supplementary Information file. Raw images and bacterial strains that support the findings of this study are available from the corresponding author upon reasonable request. The raw mRNA counts and source data underlying Figs. 4a, b, 6a, b and Supplementary Figs. 6, 7, 13 are provided as a Source Data file. The cryoEM structure in Supplementary Fig. 11 has been published by Glyde et al.^19^ and can be accessed via the RCSB Protein Data Bank (ID: 5NSS).

## Source data

Source data

## Code availability

All code used for model inference is available at the github repository (https://github.com/rdbrackston/tx-analysis*)*.
