# Supplementary material for: The route to transcription initiation determines the mode of transcriptional bursting in E. coli
Source: Nat Commun. 2020 May 15;11:2422. doi: 10.1038/s41467-020-16367-6 (PMC7229158; doi:10.1038/s41467-020-16367-6)
Supplement: Supplementary file 1 — Supplementary Information [file 41467_2020_16367_MOESM1_ESM.pdf]

Supplementary Information

The route to transcription initiation determines the mode of transcriptional bursting in *E. coli*.

Engl et al.

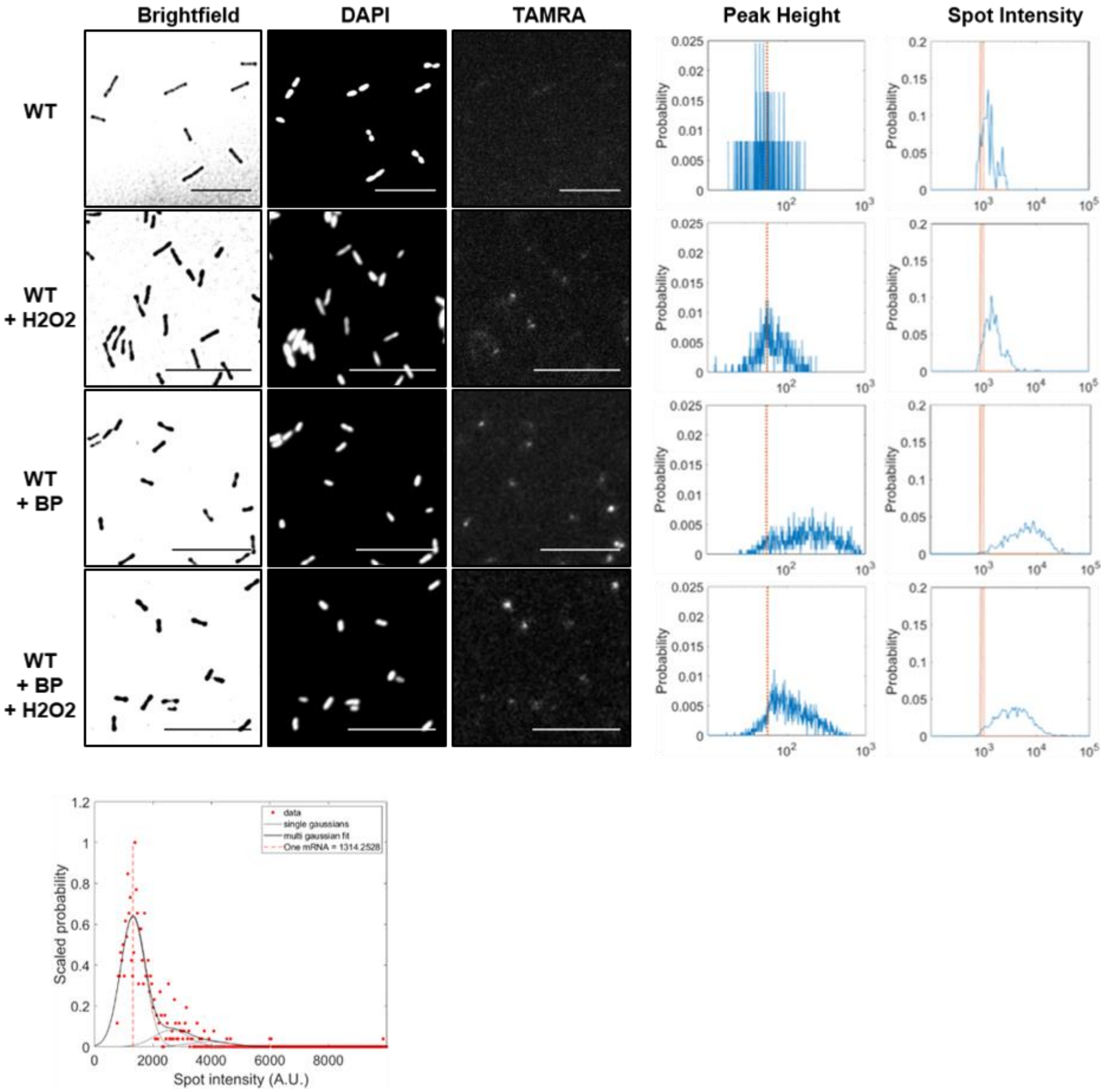

Supplementary Figure 1. Outputs from RNA Fluorescence *in situ* hybridisation to measure *sufABCD* mRNA copy numbers in wildtype (WT) *E. coli* cells. Cells were grown in absence or presence of oxidative stress (H_2O_2) and/or iron depletion (BP). The *sufABCD* mRNA was detected via 6-TAMRA fluorescently labelled DNA probes. **Upper panel**: Shown are representative micrographs from all 3 channels, the probability distributions of peak height and intensity of fluorescent spots in all cells per strain and growth condition. The data was extracted from micrographs taken with the Cy3 (TAMRA) channel. The orange line depicts the threshold to discard false positive fluorescent spots, determined via the 99.9 percentile of fluorescent spots in a negative sample that was unable to express the *sufABCD* mRNA. A total of n_{WT}= 489, $n_{WT+H2O2}$= 533, n_{WT+BP}= 529 and $n_{WT+BP+H2O2}$= 487 cells were imaged across multiple fields of view were examined from 3 independent biological replicates. Scale bar: 10 µm. **Bottom panel:** The spot intensity distribution from WT+H_2O_2 was fitted to a multiple Gaussian function to determine the spot intensity of a single *sufABCD* mRNA (mean of the first Gaussian). The value was used to measure the *sufABCD* mRNA copy number in WT, Δ*fur* and Δ*oxyR* in absence and presence of stress.

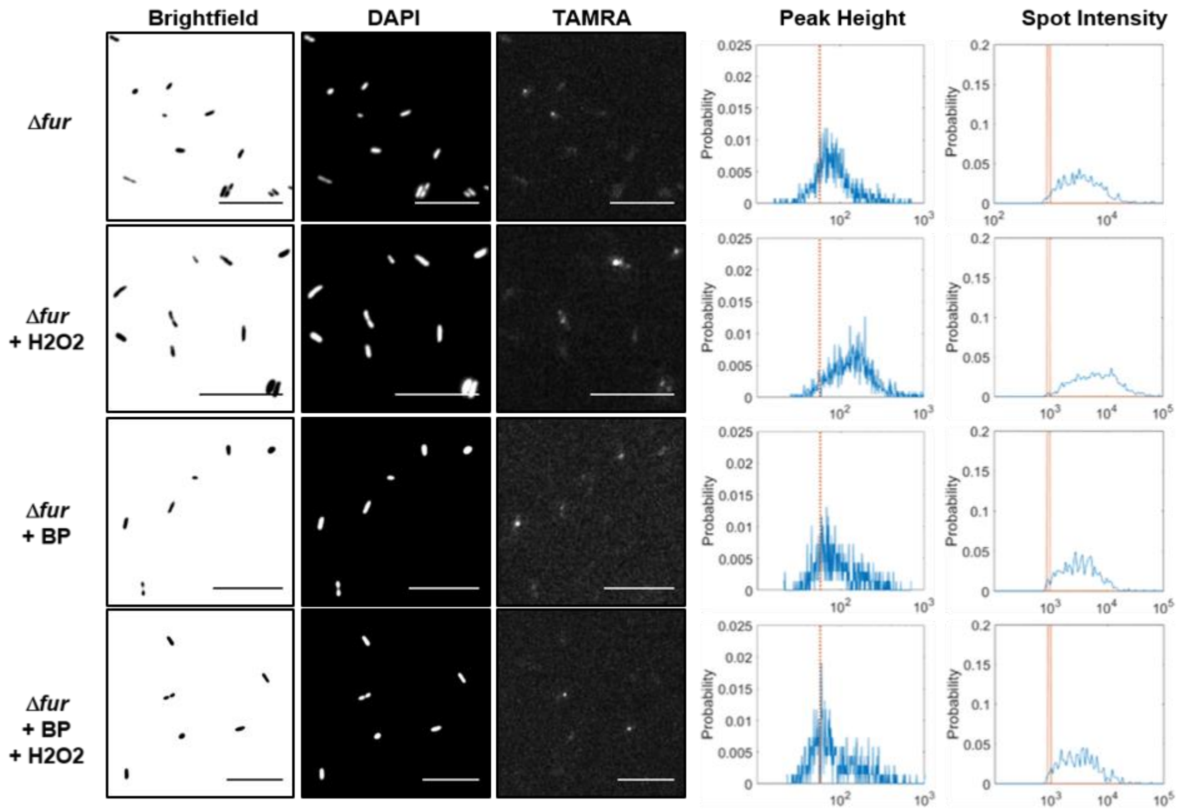

Supplementary Figure 2. Outputs from RNA Fluorescence *in situ* hybridisation to measure *sufABCD* mRNA copy numbers in *E. coli* cells lacking *fur* (Δ*fur*). Cells were grown in absence or presence of oxidative stress (H_2O_2) and/or iron depletion (BP). The *sufABCD* mRNA was detected via 6-TAMRA fluorescently labelled DNA probes. Shown are representative micrographs from all 3 channels, as well as the probability distributions of peak height and intensity of fluorescent spots in all cells per strain and growth condition. The data was extracted from micrographs taken with the Cy3 (TAMRA) channel. The orange line depicts the threshold to discard false positive fluorescent spots, determined via the 99.9 percentile of fluorescent spots in a negative sample that was unable to express the *sufABCD* mRNA. A total of $n_{\Delta fur}$ = 455, $n_{\Delta fur\text{+H2O2}}$= 667, $n_{\Delta fur\text{+BP}}$= 288 and $n_{\Delta fur\text{+BP+H2O2}}$= 365 cells across multiple fields of view were examined from 3 independent biological replicates. Scale bar: 10 μm.

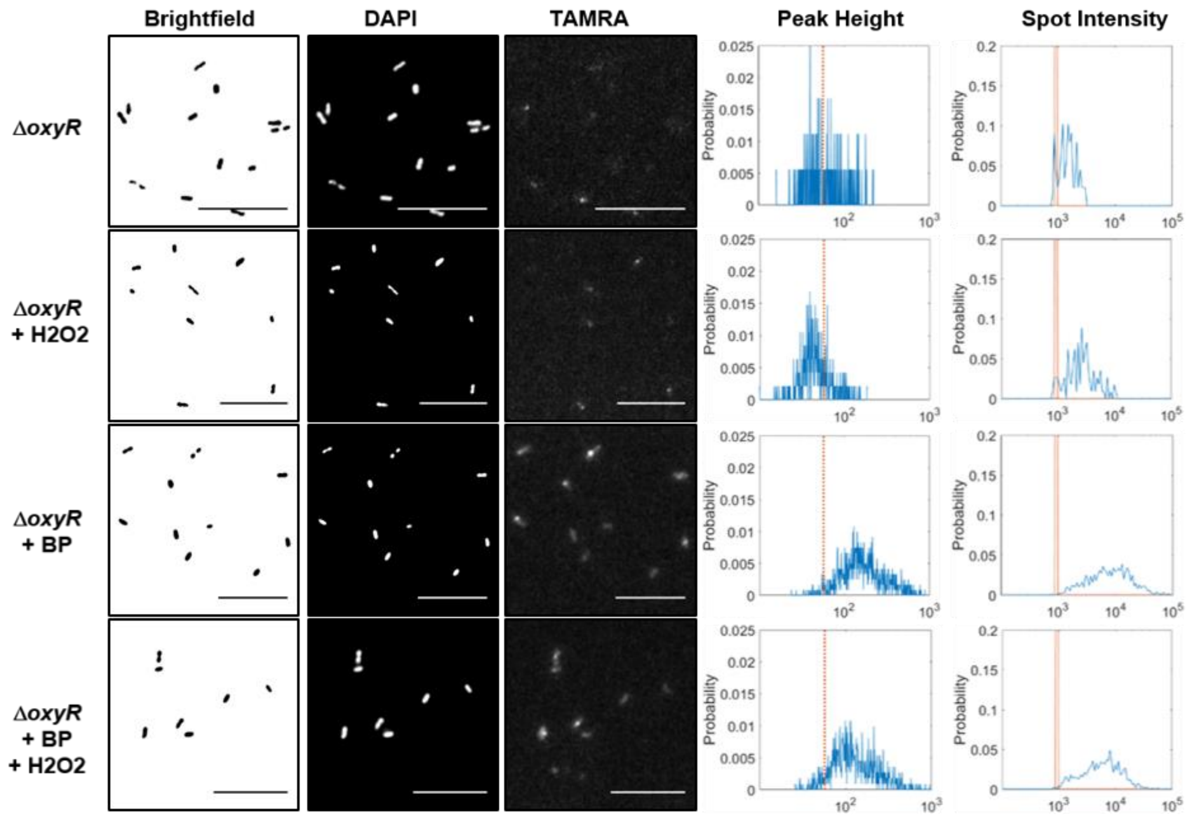

Supplementary Figure 3. Outputs from RNA Fluorescence *in situ* hybridisation to measure *sufABCD* mRNA copy numbers in *E. coli* cells lacking *oxyR* (Δ*oxyR*). Cells were grown in absence or presence of oxidative stress (H_2O_2) and/or iron depletion (BP). The *sufABCD* mRNA was detected via 6-TAMRA fluorescently labelled DNA probes. Shown are representative micrographs from all 3 channels, as well as the probability distributions of peak height and intensity of fluorescent spots in all cells per strain and growth condition. The data was extracted from the micrographs taken with the Cy3 (TAMRA) channel. The orange line depicts the threshold to discard false positive fluorescent spots, determined via the 99.9 percentile of fluorescent spots in a negative sample that was unable to express the *sufABCD* mRNA. A total of $n_{\Delta oxyR}$ = 357, $n_{\Delta oxyR\text{+H2O2}}$= 379, $n_{\Delta oxyR\text{+BP}}$= 494 and $n_{\Delta oxyR\text{+BP+H2O2}}$= 510 cells across multiple fields of view were examined from 3 independent biological replicates. Scale bar: 10 μm.

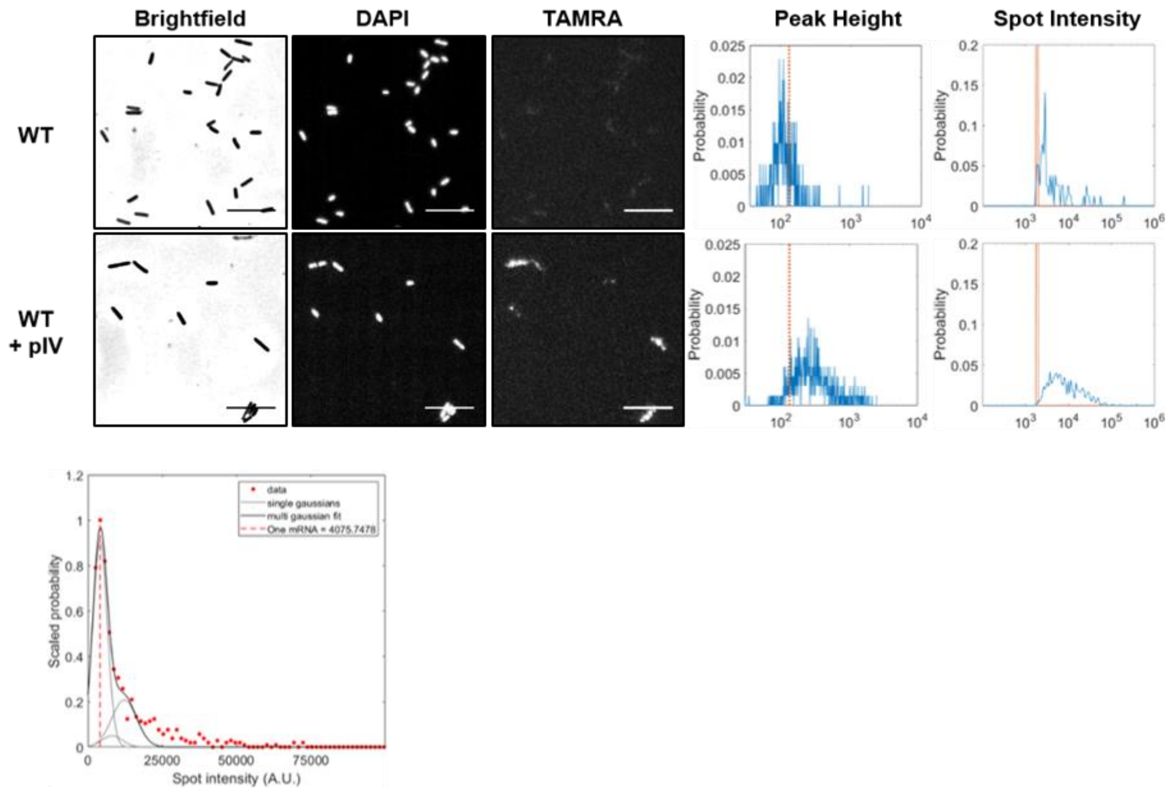

Supplementary Figure 4. Outputs from RNA Fluorescence *in situ* hybridisation to measure *pspABC* mRNA copy numbers in wildtype (WT) *E. coli* cells. Cells were grown in absence and presence of inner membrane stress (pIV). The *pspABC* mRNA was detected via 6-TAMRA fluorescently labelled DNA probes. **Upper panel**: Shown are representative micrographs from all 3 channels, the probability distributions of peak height and intensity of fluorescent spots in all cells per strain. The data was extracted from micrographs taken with the Cy3 (TAMRA) channel. The orange line depicts the threshold to discard false positive fluorescent spots, determined via the 99.9 percentile of fluorescent spots in a negative sample that was unable to express the *pspABC* mRNA. A total of n_{WT} = 437 and n_{WT+pIV}= 202 cells across multiple fields of view were examined from 3 independent biological replicates. Scale bar: 10 μm. **Bottom panel:** The spot intensity distribution from WT+pIV was fitted to a multiple Gaussian function to determine the spot intensity of a single *pspABC* mRNA (mean of the first Gaussian). The value was used to measure the *pspABC* mRNA copy number in all WT cells grown in absence and presence of pIV.

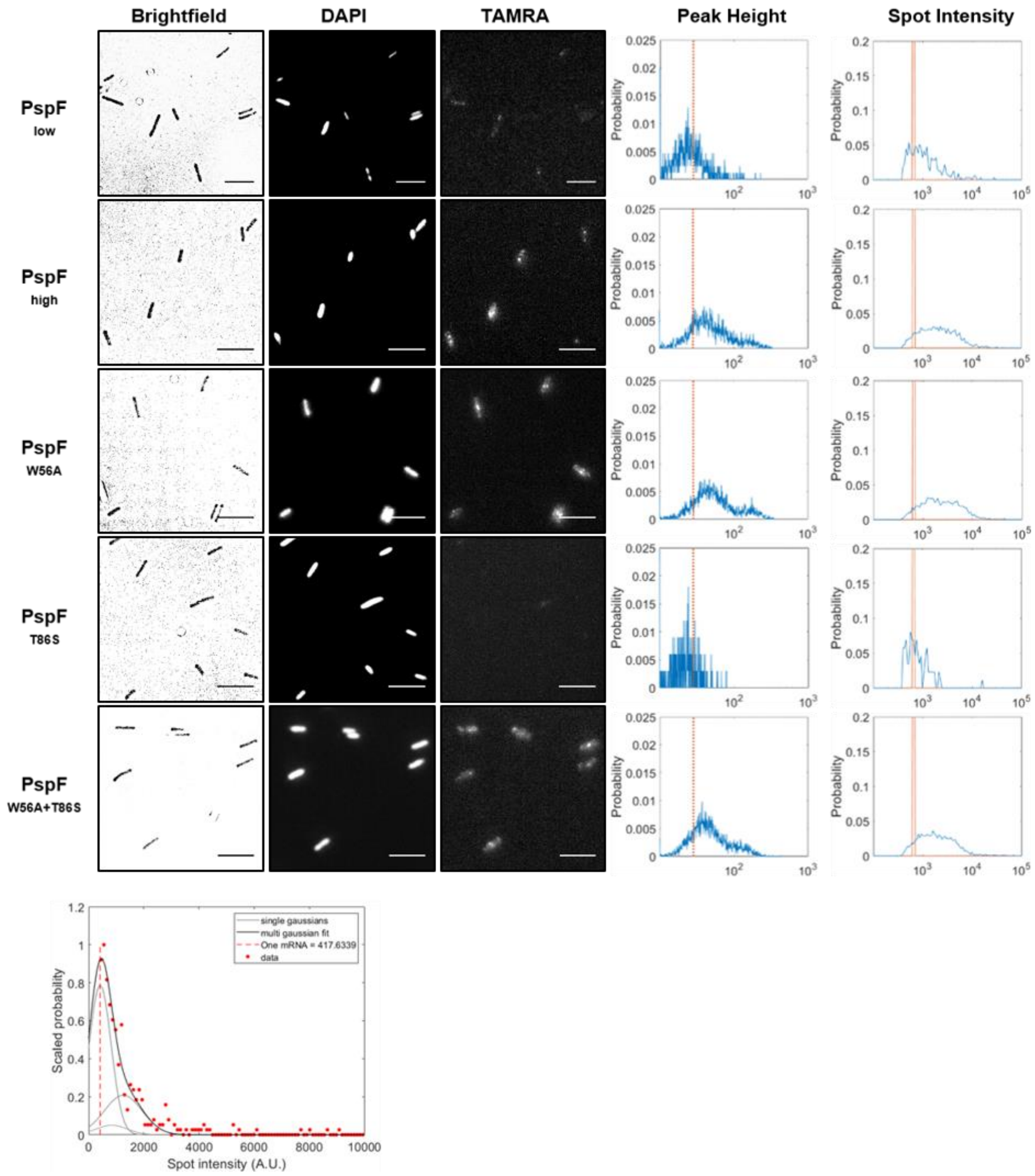

Supplementary Figure 5. Outputs from RNA Fluorescence *in situ* hybridisation to measure *pspABC* mRNA copy numbers in *E. coli* cells with heterologous PspF expression. Cells expressed wildtype PspF at low or high levels. PspF variants were expressed at low levels. The *pspABC* mRNA was detected via 6-TAMRA fluorescently labelled DNA probes. **Upper panel**: Shown are representative micrographs from all 3 channels, the probability distributions of peak height and intensity of fluorescent spots in all cells per strain. The data was extracted from micrographs taken with the Cy3 (TAMRA) channel. The orange line depicts the threshold to discard false positive fluorescent spots, determined via the 99.9 percentile of fluorescent spots in a negative sample that was unable to express the *pspABC* mRNA. A total of n_{PspFlow}= 496, n_{PspFhigh}= 416, n_{PspFW56A}= 389, n_{PspFT86S}= 452 and $n_{\text{PspFW56A+T86S}}$= 432 cells across multiple fields of view were examined from 3 independent biological replicates. Scale bar: 10 µm. **Bottom panel:** The spot intensity distribution from PspF_{low} was fitted to a multiple Gaussian function to determine the spot intensity of a single *pspABC* mRNA (mean of the first Gaussian). The value was used to measure the *pspABC* mRNA copy number in all cells with heterologous PspF expression.

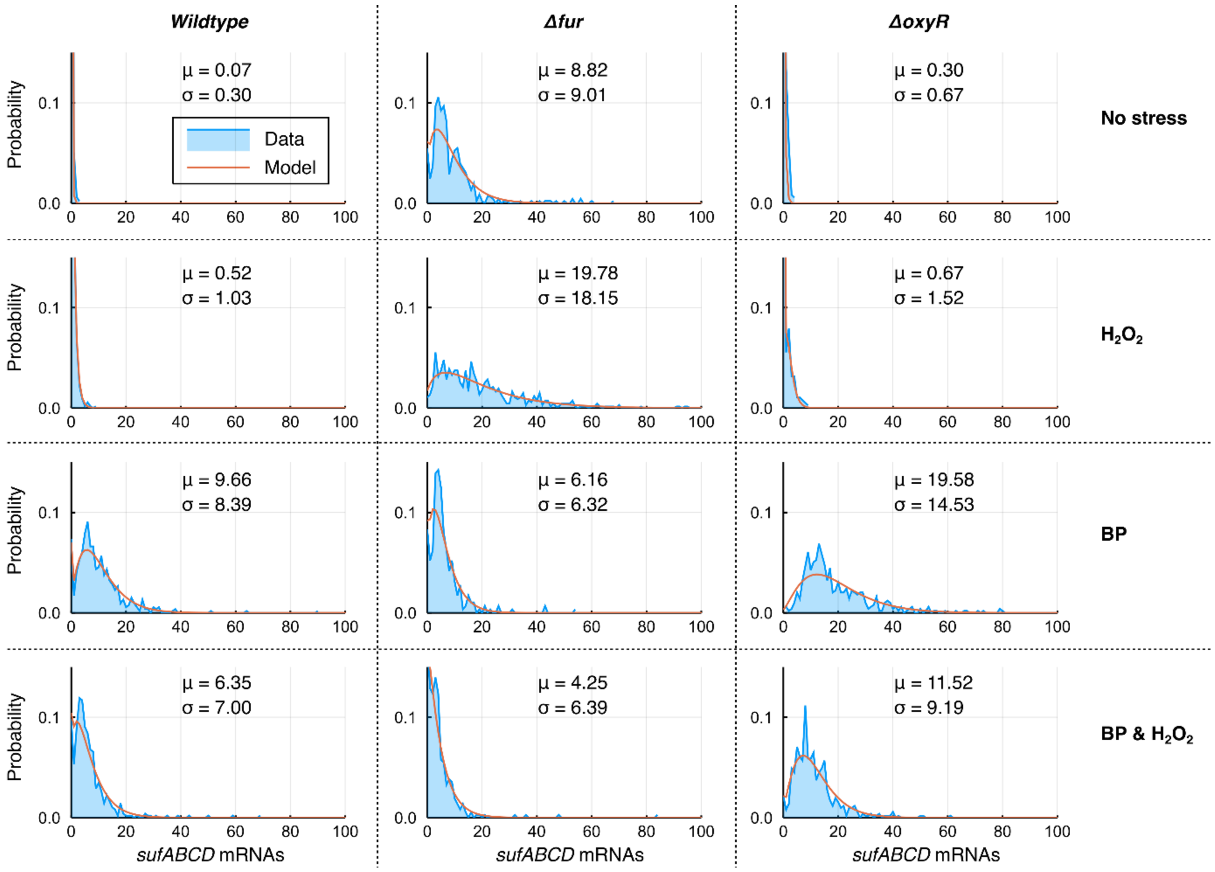

Supplementary Figure 6. Number of *sufABCD* mRNAs per cell. Shown is the probability distributions of *sufABCD* mRNAs per cell**,** the model fit (red solid line) as well as mean (μ) and standard deviation (σ). The graphs are arranged by strain (vertical) and stress condition (horizontal).

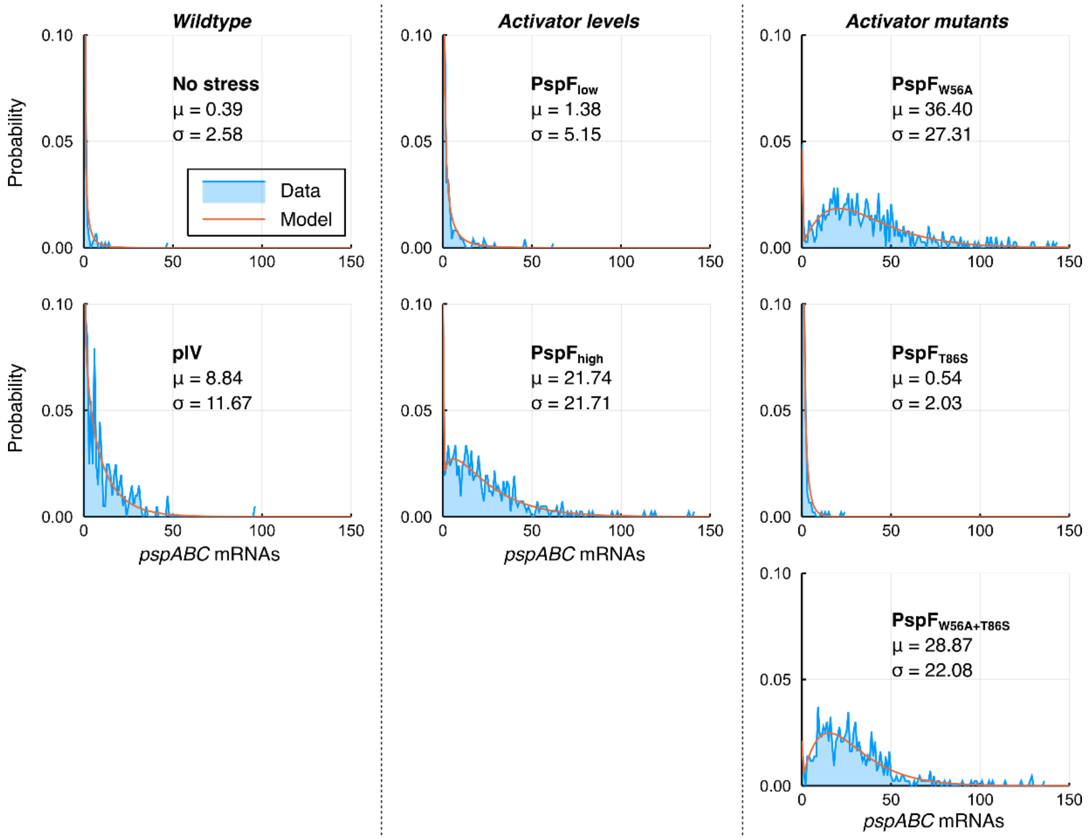

Supplementary Figure 7. Number of *pspABC* mRNAs per cell. Shown is the probability distributions of *pspABC* mRNAs per cell**,** the model fit (red solid line) as well as mean (μ) and standard deviation (σ). The graphs are arranged vertically into no stress vs stress (Wildtype; left panel), expression level of wildtype PspF (Activator levels; middle panel) and PspF variants (Activator mutants; right panel).

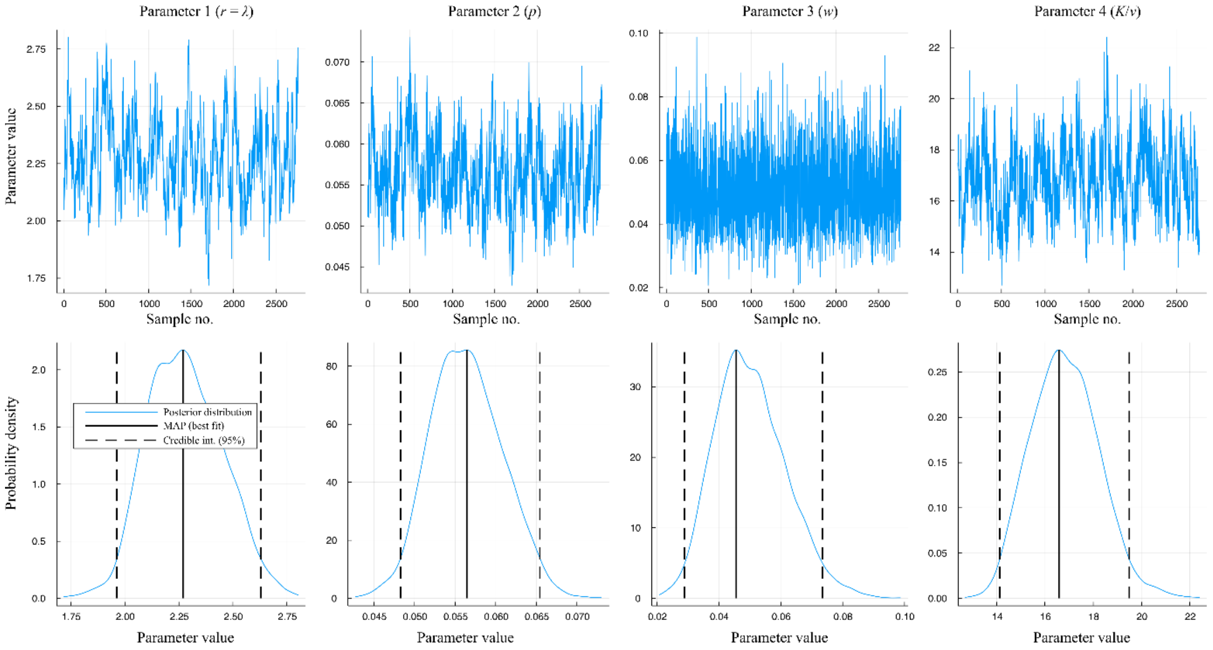

Supplementary Figure 8. An example Markov chain Monte Carlo sample produced during the parameter inference process. Chains were run for 300,000 iterations before being truncated and thinned. Parameter estimates were taken as the maximum *a posteriori* (MAP) given by the peak of the posterior distribution. Error bounds on the parameter estimates were obtained as the 95% Bayesian credible intervals.

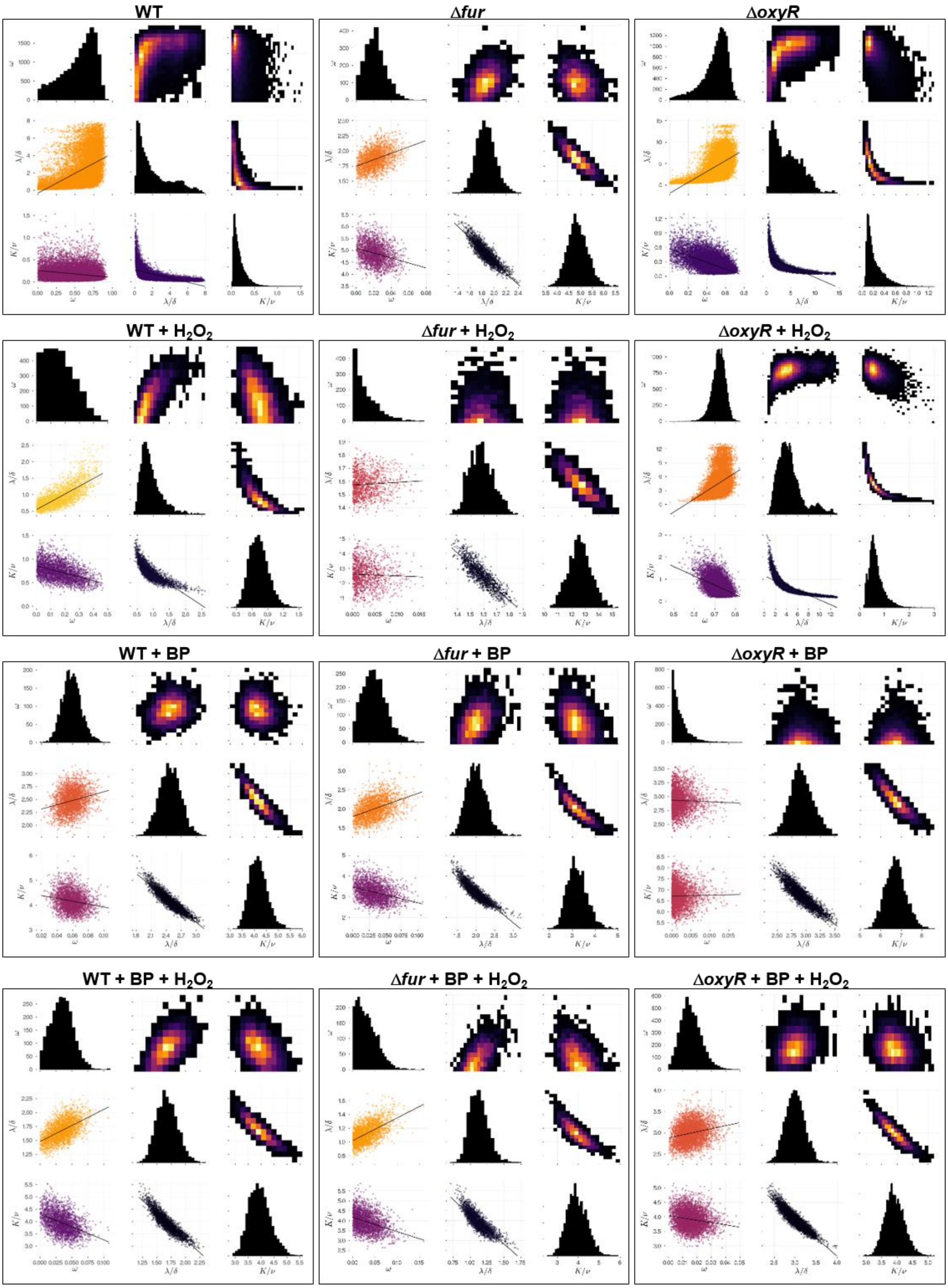

Supplementary Figure 9. Outputs from Markov chain Monte Carlo sampling for the analysis of σ^{70} controlled transcription from the *Suf* promoter. Shown are corner plots for each of the Markov chain Monte Carlo chains displaying the posterior distributions for the inferred parameters as well as the dependencies between those parameters.

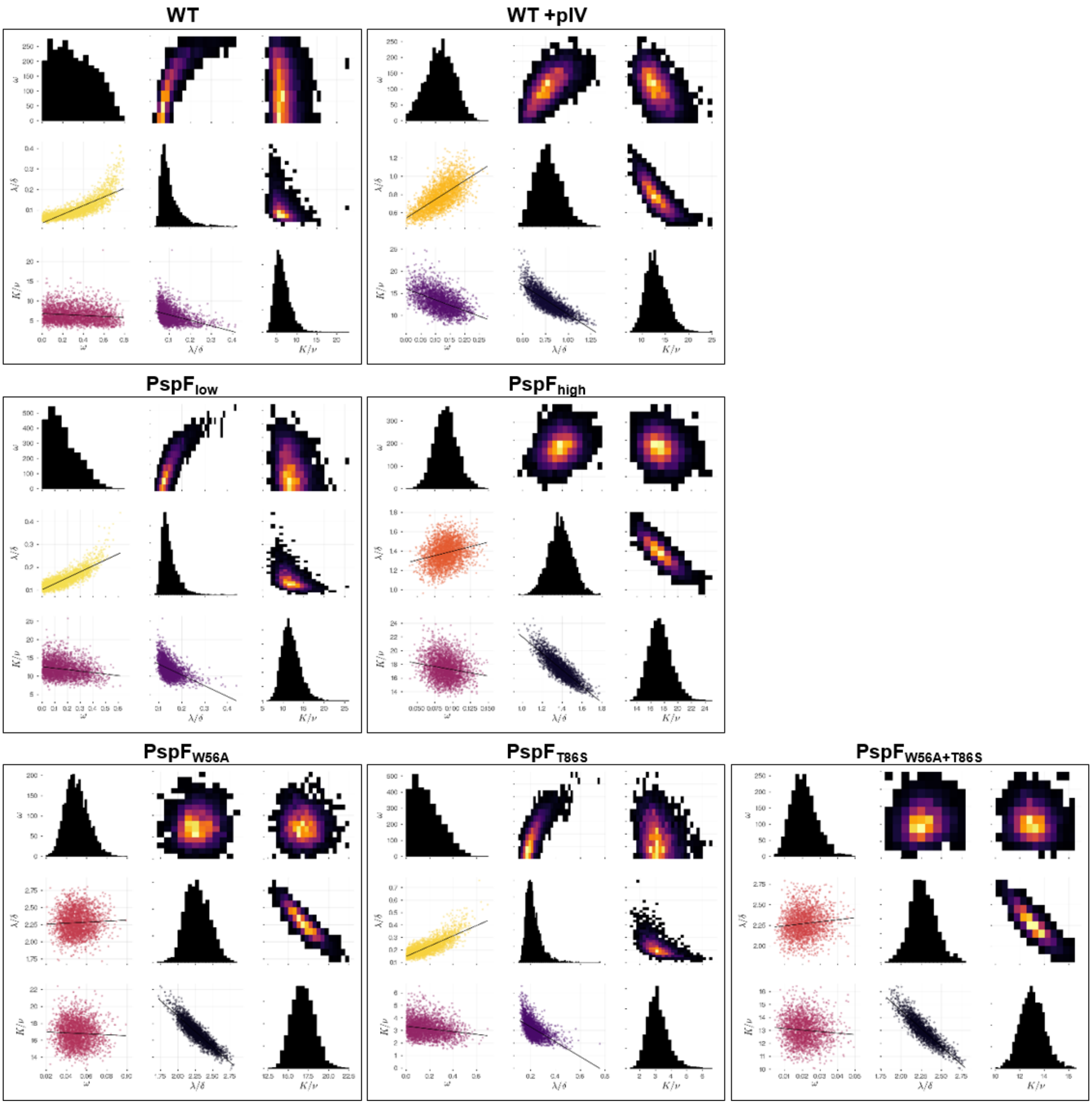

Supplementary Figure 10. Outputs from Markov chain Monte Carlo sampling for the analysis of σ^{54} controlled transcription from the *Psp* promoter. Shown are corner plots for each of the Markov chain Monte Carlo chains displaying the posterior distributions for the inferred parameters as well as the dependencies between those parameters.

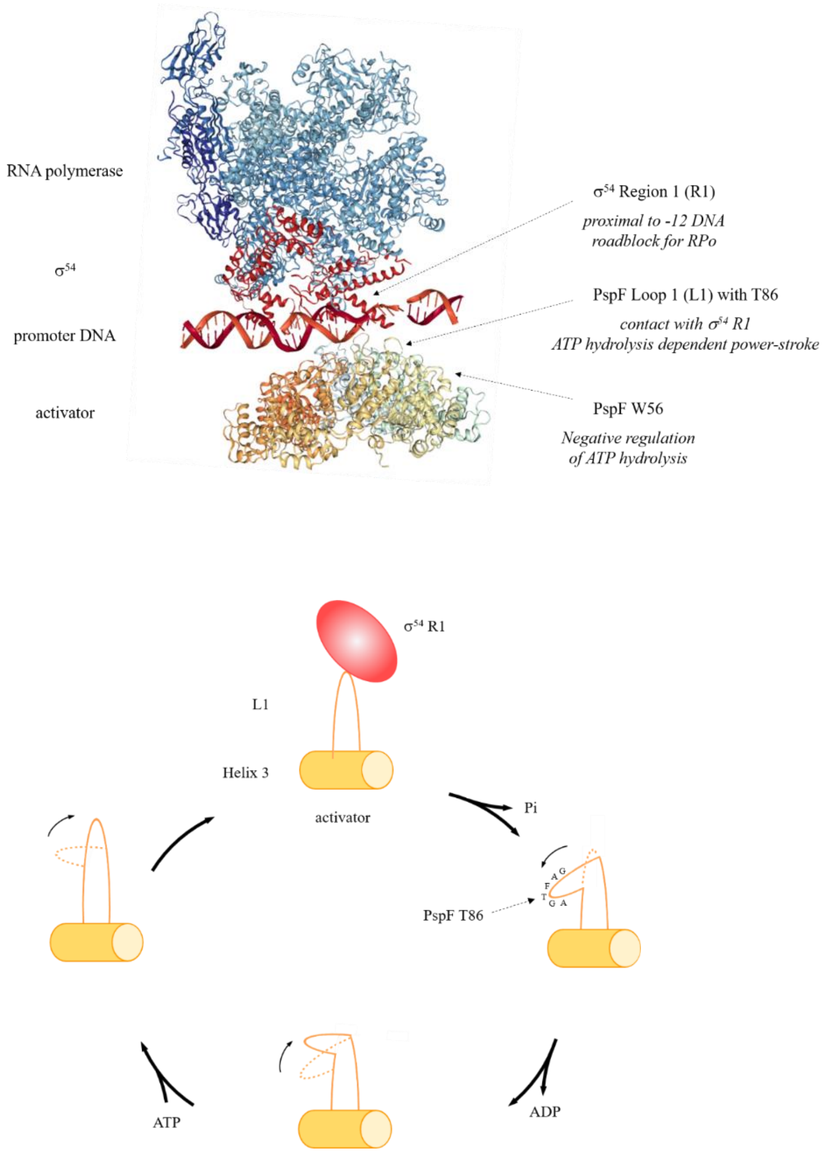

Supplementary Figure 11. Molecular detail of the interaction between activator PspF and RNA polymerase/σ^{54}. Upper panel: Cryo-EM structure (RCSB Protein Data Bank ID: 5NSS) of the intermediate complex between RNA polymerase/σ^{54} with promoter DNA and activator PspF. The structure is published in Supplementary Reference 2. Region 1 (R1) of σ^{54} is located proximal to the -12 promoter DNA and acts as a roadblock for open complex (RPo) formation. T86 is located within Loop 1 (L1) of PspF. L1 of PspF is in contact with R1 of σ^{54}. The T86S mutation weakens the contact between L1 of PspF and R1 of σ^{54}. The location of W56, the site of negative regulation of PspF ATP hydrolysis by PspA, is indicated. The W56A mutation prevents interaction of PspA and PspF and thus negative regulation. $PspF_{W56A}$ is constitutively active for ATP hydrolysis. **Bottom panel:** L1 of PspF performs a ATP-hydrolysis dependent powerstroke to contact and move R1 of σ^{54}. This movement enables open complex formation and thus transcription initiation. The schematic is adapted from Supplementary Reference 3.

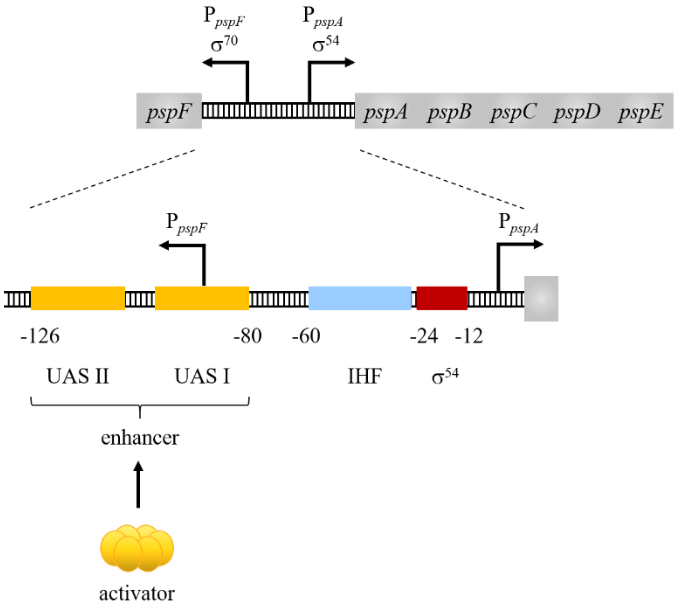

Supplementary Figure 12. Autoregulation of activator expression in the native *Psp* system of *E. coli*. Shown is the *pspABCDE* operon and the adjacent divergently transcribed monocistronic *pspF* gene. The operon is regulated by the σ^{54} controlled P_{pspA} promoter and encodes the negative regulator and effector PspA, the sensors of the stress signal PspBC, the effector PspD and the rhodanese PspE. Expression of *pspF* is under the control of the σ^{70}-dependent promoter P_{pspF}. The activator PspF binds to Upstream Activator Sequences (UAS I and II), also termed enhancer to initiate transcription of the *pspABCDE* operon. These are located around 100 nucleotides upstream of the transcriptional start site and overlap with the P_{pspF} promoter. Binding of PspF to the enhancer thus results in its negative autoregulation yielding consistently low levels of intracellular PspF levels. Located between -25 and -60 are binding sites for Integration Host Factor (IHF). IHF causes looping of the DNA that brings the enhancer-bound activator PspF in contact with the RNA polymerase-σ^{54} at the -24/-12 site. The schematic is adapted from Supplementary Reference 4.

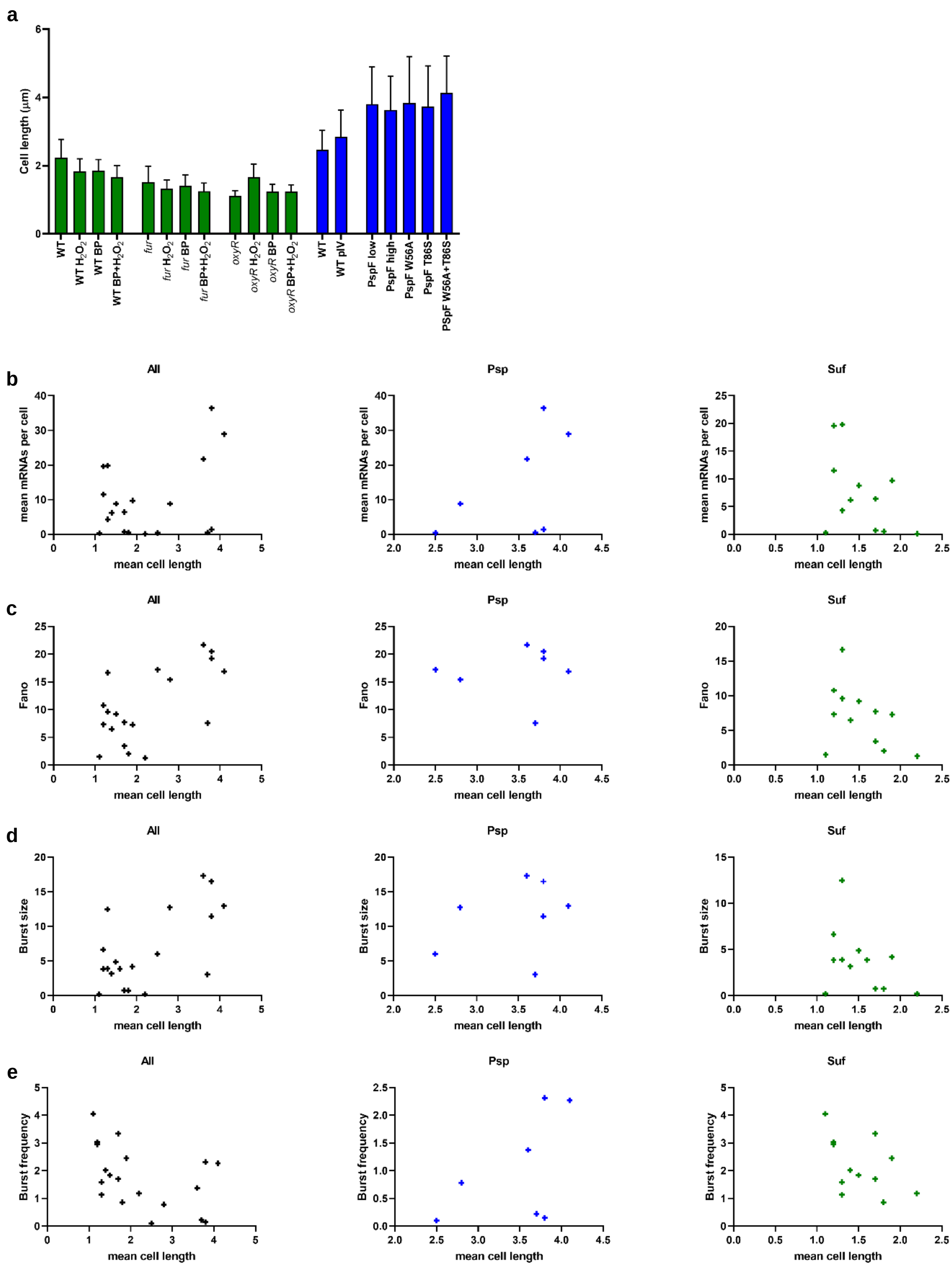

Supplementary Figure 13. Correlation between cell length and burst parameters. The length of each cell per strain and condition tested (green bars: *Suf*; blue bars: *Psp*) was extracted as part of the Spätzcells analysis. **(a)** Shown is the mean cell length and error bars depict SD. The mean cell length was plotted against **(b)** mean mRNAs per cell, **(c)** Fano factor, **(d)** Burst size and **(e)** Burst frequency. For *Suf* a total of n_{WT}= 489, $n_{WT+H2O2}$= 533, n_{WT+BP}= 536, $n_{WT+BP+H2O2}$= 373, $n_{\Delta fur}$= 460, $n_{\Delta fur+H2O2}$= 674, $n_{\Delta fur+BP}$= 290, $n_{\Delta fur+BP+H2O2}$= 369, $n_{\Delta oxyR}$= 357, $n_{\Delta oxyR+H2O2}$= 379, $n_{\Delta oxyR+BP}$= 499, $n_{\Delta oxyR+BP+H2O2}$= 514 cells from 3 independent biological replicates were examined. For *Psp* a total of n_{WT}= 438, n_{WT+pIV}= 205, $n_{PspFlow}$= 497, $n_{PspFhigh}$= 467, $n_{PspFW56A}$= 437, $n_{PspFT86S}$= 262 and $n_{PspFW56A+T86S}$= 480 cells from 3 independent biological replicates were examined.

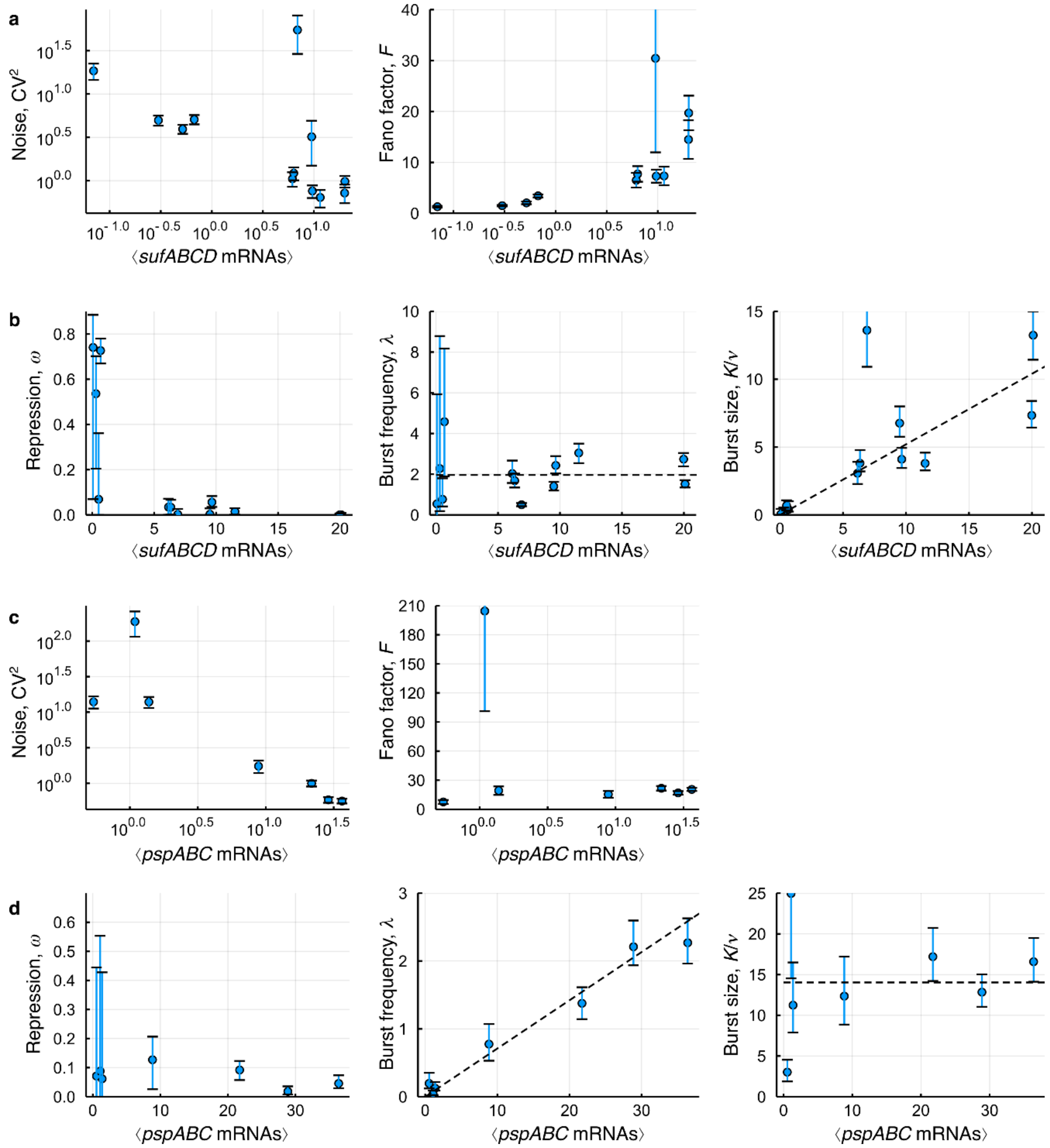

Supplementary Figure 14. Single cell gene expression data without applying a cut-off. Shown is the noise, burstiness (Fano factor) and burst kinetics of transcription from **(a, b)** P_{sufA} and **(c, d)** P_{pspA}. The data shows a similar trend than when a cut-off at 150 mRNAs per cell is applied, see main text. Data in **(a)** and **(c)** are presented as the statistic of the full set of data samples +/- SEM obtained from *n*=10000 bootstrap resamples. Data in **(b)** and **(d)** are presented as the maximum a posteriori parameter estimates and error bars are 95% credible intervals, both derived from the posterior distributions of the Markov chain Monte Carlo sampling.

Supplementary Note 1.

Can the differences in transcriptional burst kinetic modulations at σ^{70} and σ^{54} promoters be explained by differences in gene dosage and RNA lifetime?

Differences in gene dosage during cell cycle. As suggested[1], cells were grown in M9 minimal medium to reduce growth rate and thus limit chromosome replication and associated gene dosage effects. Moreover, when generating masks prior to Spätzcells analysis, cells were inspected by eye for cell division and any apparent chromosome replication. Masks of cells with a clear invagination and more than one discernible nucleoid (as judged by DAPI staining) were either removed or adjusted to yield one nucleoid per mask. As previously described[1,5], we have measured cell length to further account for potential gene copy number effects and plotted cell length against mean mRNA copy number, Fano factor, burst size and burst frequency (Supplementary Fig. 13). For both *Suf* and *Psp*, cell length of untreated WT cells was similar. For *Suf*, cell length of Δ*fur*, Δ*oxyR* with & without BP and/or H_2O_2 was reduced compared to untreated WT cells. For *Psp*, cell length upon heterologous PspF expression was similar; yet larger than WT and WT pIV. Although cell length of *Suf* WT and *Psp* WT is similar, their respective Fano factor, burst size and burst frequency are not (Figs. 4, 6, Supplementary Fig. 13).

The same is observed for cells with heterologous expression of PspF. Despite similar cell length, their Fano factor (see $PspF_{T86S}$ vs all other PspF strains), burst size (see $PspF_{T86S}$ vs all other PspF strains) and burst frequency (see $PspF_{low}$ vs $PspF_{high}$, $PspF_{T86S}$ vs $PspF_{high}$, $PspF_{W56A}$ and $PspF_{W56A+T86S}$) differ markedly (Fig 6; Supplementary Fig. 13). Thus in our data set, we did not observe a clear correlation between cell length (and by inference gene copy number) and either mean mRNA copy number, Fano, burst size or burst frequency (Supplementary Fig. 13).

Overall, the magnitude of burst frequency modulation seen at the Psp locus is far greater than could be accounted for by the simple linear relationship that exists between burst frequency and gene copy number for a number of σ^{70} promoters, as reported[5]. We therefore conclude that the differences in burst parameter modulations are indeed due to the differences in sigma factors and not simply due to differences in gene dosage during the cell cycle.

Differences in RNA lifetime. A recent study found no correlation between gene function and RNA lifetime[6]. Instead, RNA lifetime is dependent on transcript intrinsic features[7]. A change in RNA lifetime of a transcript thus requires a change in growth condition e.g. cell cycle. Our PspF expressing strains were grown to exponential phase in the same medium and reside in a similar cell cycle (as judged by the cell length). We therefore infer that the conditions for RNA degradation and thus RNA lifetime are the same; yet their burst parameters differ. Hence, although in principle possible, it is unlikely that RNA lifetime changes can explain our observations. Instead, we conclude that the differences in mRNA copy numbers are due to transcription initiation. Indeed, it was reported that changes in rates of transcription initiation provide a better explanantion for change in RNA abundance than changes in RNA lifetimes[6].

Supplementary References.

1. Skinner, S.O., Sepúlveda, L.A., Xu, H. & Golding, I. Measuring mRNA copy number in individual Escherichia coli cells using single-molecule fluorescent in situ hybridization. *Nat. Protoc.* **8**, 1100–1113 (2013).

2. Glyde, R., Ye, F., Darbari, V.C., Zhang, N., Buck, M. & Zhang, X. Structures of RNA Polymerase Closed and Intermediate Complexes Reveal Mechanisms of DNA Opening and Transcription Initiation. *Mol. Cell* **67**, 106–116.e4 (2017).

3. Bush, M. & Dixon, R. The role of bacterial enhancer binding proteins as specialized activators of σ54-dependent transcription. *Microbiol. Mol. Biol. Rev.* **76,** 497-529 (2012).

4. Joly, N., Engl, C., Jovanovic, G., Huvet, M., Toni, T., Sheng, X., Stumpf, M.P. & Buck, M. Managing membrane stress: the phage shock protein (Psp) response, from molecular mechanisms to physiology. *FEMS Microbiol. Rev.* **34**, 797-827 (2010).

5. So, L.H., Ghosh, A., Zong, C., Sepúlveda, L.A., Segev, R., Golding, I. General properties of transcriptional time series in Escherichia coli. *Nat. Genet.* **43,** 554-60 (2011).

6. Chen H., Shiroguchi K., Ge H., Xie X.S. Genome-wide study of mRNA degradation and transcript elongation in Escherichia coli. *Mol. Syst. Biol.* **11**, 781 (2015).

7. Hui M.P., Foley P.L., Belasco J.G. Messenger RNA degradation in bacterial cells. *Annu. Rev. Genet.* **48**, 537-59 (2014).
