# Supplementary material for: The route to transcription initiation determines the mode of transcriptional bursting in E. coli
Source: Nat Commun. 2020 May 15;11:2422. doi: 10.1038/s41467-020-16367-6 (PMC7229158; doi:10.1038/s41467-020-16367-6)
Supplement: Supplementary file 2 — Reporting Summary [file 41467_2020_16367_MOESM2_ESM.pdf]

nature research

Corresponding author(s): Christoph Engl & Martin Buck

Last updated by author(s): Apr 15, 2020

Reporting Summary

Nature Research wishes to improve the reproducibility of the work that we publish. This form provides structure for consistency and transparency in reporting. For further information on Nature Research policies, see Authors & Referees and the Editorial Policy Checklist .

Statistics

For all statistical analyses, confirm that the following items are present in the figure legend, table legend, main text, or Methods section.

n/a	Confirmed	
☐	☒	The exact sample size (*n*) for each experimental group/condition, given as a discrete number and unit of measurement
☐	☒	A statement on whether measurements were taken from distinct samples or whether the same sample was measured repeatedly
☒	☐	The statistical test(s) used AND whether they are one- or two-sided<br>*Only common tests should be described solely by name; describe more complex techniques in the Methods section.*
☒	☐	A description of all covariates tested
☒	☐	A description of any assumptions or corrections, such as tests of normality and adjustment for multiple comparisons
☐	☒	A full description of the statistical parameters including central tendency (e.g. means) or other basic estimates (e.g. regression coefficient) AND variation (e.g. standard deviation) or associated estimates of uncertainty (e.g. confidence intervals)
☒	☐	For null hypothesis testing, the test statistic (e.g. *F*, *t*, *r*) with confidence intervals, effect sizes, degrees of freedom and *P* value noted<br>*Give P values as exact values whenever suitable.*
☐	☒	For Bayesian analysis, information on the choice of priors and Markov chain Monte Carlo settings
☒	☐	For hierarchical and complex designs, identification of the appropriate level for tests and full reporting of outcomes
☒	☐	Estimates of effect sizes (e.g. Cohen's *d*, Pearson's *r*), indicating how they were calculated

Our web collection on statistics for biologists contains articles on many of the points above.

Software and code

Policy information about availability of computer code

Data collection	The RNA fluorescence in situ hybridization experiments were performed as described in Skinner et al (2013) using the Matlab (Mathworks) scripts Schnitzcells (Young et al 2012) and Spatzcells (Skinner et al 2013).
Data analysis	Measurement of fluorescence intensities of single mRNAs, calculation of mRNAs per cell and estimation of transcription kinetics based on population statistics was done via the Spatzcells software (Skinner et al 2013). Model fitting was performed via a Bayesian inference approach using Markov chain Monte Carlo sampling using our own code. All code used for model inference is available at the github repository (https://github.com/rdbrackston/tx-analysis).

For manuscripts utilizing custom algorithms or software that are central to the research but not yet described in published literature, software must be made available to editors/reviewers. We strongly encourage code deposition in a community repository (e.g. GitHub). See the Nature Research guidelines for submitting code & software for further information.

Data

Policy information about availability of data

All manuscripts must include a data availability statement. This statement should provide the following information, where applicable:

- Accession codes, unique identifiers, or web links for publicly available datasets
- A list of figures that have associated raw data
- A description of any restrictions on data availability

The authors declare that the data supporting the findings of this study are available within the paper and its Supplementary Information file. Raw images and bacterial strains that support the findings of this study are available from the corresponding author upon reasonable request. The raw mRNA counts and source data underlying Figs. 4a, 4b, 6a, 6b and Supplementary Figs. 6, 7, 13 are provided as a Source Data file. The cryoEM structure in Supplementary figure 11 is published by Glyde et al (2017) and can be accessed via the RCSB Protein Data Bank (ID: 5NSS).

Field-specific reporting

Please select the one below that is the best fit for your research. If you are not sure, read the appropriate sections before making your selection.

- [x] Life sciences
- [ ] Behavioural & social sciences
- [ ] Ecological, evolutionary & environmental sciences

For a reference copy of the document with all sections, see nature.com/documents/nr-reporting-summary-flat.pdf

Life sciences study design

All studies must disclose on these points even when the disclosure is negative.

Sample size	We imaged multiple fields of view per strain and condition tested and collected images of several hundred cells, with the particular number in each case limited by practical considerations. Our minimal sample size was 202 but for most experiments we imaged more than 400 cells. No statistical methods were used to determine sample sizes, but the effect of sample size is taken into account in the analysis through the calculation of the displayed confidence intervals.
Data exclusions	We mention in the Mathematical modelling and computational analysis section of the Methods: We calculated noise, burstiness and burst kinetics from a population of cells expressing up to 150 mRNAs of the analysed genes. This is based on the available in vitro data on the activity of s54 promoters showing that each open promoter complex 1-2 minutes to form. During one cell division (~30 minutes) we assume therefore that 50 mRNAs per cell would be towards the top end of what might accumulate if the usual parameters of promoter activity and mRNA stability were being met. We note that some cells appeared to accumulate several hundreds of target mRNAs. We propose that these high content mRNA cells are imaging or hybridisation artefacts eg through probe aggregation or non-specific genome wide hybridisation. These cells were therefore omitted from the analyses presented in the main text. For completeness, we have included the data derived from the analyses including these cells in the Supplementary Information. The overall trends of noise, burstiness and burst kinetics were similar to the range of 0-150 mRNAs per cell.
Replication	Samples were from 3 independent biological replicates and the experimental findings were reliably reproduced.
Randomization	Randomization was not relevant to this study because samples were not allocated to groups.
Blinding	Blinding was not relevant to this study since there was no group allocation.

Reporting for specific materials, systems and methods

We require information from authors about some types of materials, experimental systems and methods used in many studies. Here, indicate whether each material, system or method listed is relevant to your study. If you are not sure if a list item applies to your research, read the appropriate section before selecting a response.

Materials & experimental systems

n/a	Involved in the study
x	[ ] Antibodies
x	[ ] Eukaryotic cell lines
x	[ ] Palaeontology
x	[ ] Animals and other organisms
x	[ ] Human research participants
x	[ ] Clinical data

Methods

n/a	Involved in the study
x	[ ] ChIP-seq
x	[ ] Flow cytometry
x	[ ] MRI-based neuroimaging
